# High-resolution weather network reveals a high spatial variability in air temperature in the Central valley of California with implications for crop and pest management

**DOI:** 10.1371/journal.pone.0267607

**Published:** 2022-05-19

**Authors:** Johann Martínez-Lüscher, Tomas Teitelbaum, Anthony Mele, Oliver Ma, Andrew Jordan Frewin, Jordan Hazell

**Affiliations:** Semios Incorporated, Great Northern Way Vancouver, Vancouver, BC, Canada; Soil and Water Resources Institute ELGO-DIMITRA, GREECE

## Abstract

Weather is the most important driver of crop development. However, spatial variability in weather makes it hard to obtain reliable high resolution datasets across large areas. Most growers rely on data from a single station that can be up to 50km away to make decisions about irrigation, pest management and penology-associated cultural practices at the block level. In this regard, we hypothesize that kriging a large network of weather stations can improve thermal time data quality compared to using the closest station. This study aims to explore the spatial variability in California’s Central Valley and what is the relationship between the density of weather stations used and the error in the measurement of temperature related metrics and derived models. For this purpose, we used temperature records from January 1st 2020 to March 1st 2021 collected by the California Irrigation Management Information System (CIMIS) and a system of 731 weather stations placed above the canopy of trees in commercial orchards (in-orchard). We observed large discrepancies (>300 GDD_Tb0_) in thermal time accumulation between using an interpolation of all stations available and just using the closest CIMIS station. Our data suggests these differences are not systematic bias but true differences in mesoclimate. Similar results were observed for chill accumulation in areas especially prone to not meeting pistachio chill requirements where the discrepancies between using the site-specific in-orchard weather station network and not using them were up to 10 CP. The use of this high resolution network of weather stations revealed spatial patterns in grape, almond, pistachio and pests phenology not reported before. Whereas previous studies have been focused on predictions at the county or state or regional level, our data suggests that a finer resolution can result in major improvements in the quality of data crucial for crop decision making.

## 1. Introduction

Temperature records have a great utility in agriculture as they enable the calculation of thermal time. Ectothermic organisms such as plants, and most pests and pathogens, have a metabolism regulated by their ambient temperature. These organisms have a minimum temperature to become active and above that threshold their rate of growth, development and reproduction increases. Although each organism may have a different base temperature and adjusting this can help to refine models [1], the growing degree days (GDD) model is the most widely used system of measuring thermal time. The accumulation of thermal time is strongly related to crop phenology, including budbreak, flowering, vegetative growth, fruit development and senescence [2, 3]; and insect development and phenology, including when a pest may emerge and how many generations may occur in a season [4]. Temperature records and thermal time are also valuable for the modeling of bacterial and fungal pests, however, they also rely heavily on the use of precipitation and relative humidity records [5].

There are a great number of examples where GDD can be used to make informed decisions. For instance, the selection of grape varieties is greatly linked to the average thermal time accumulated at a specific location [6]. Similar needs for weather data analysis exist with other crops if they require a long growing season, have a chill requirement, or absence of frosts [7]. Growing degree days are the main input of many agricultural models that aim to predict the optimal time to implement pest management. This is the case of almonds or pistachios that become susceptible to navel orangeworm, *Amyelois transitella* (Lepidoptera: Pyralidae), at hull split. Therefore, it is important to time navel orangeworm pest management around this physiological plant stage in order to minimize potential losses [8]. In addition, in the control of navel orangeworm itself it is important to be able to predict the resident population’s phenology to optimize the application of chemical management strategies and mating disruption resources accordingly.

Plant development has proven to be very related to warm temperatures as long as water and nutrients are maintained within reasonable limits and a simple model as GDD is enough in most cases. However, this is not the case for bud dormancy. Many tree species, including most tree crops, have a chill requirement that must be met in order to resume normal growth and flower after winter dormancy. Although early efforts to understand chill requirements such as the accumulation of chilling hours (hours from 0 to 7.2°C; [9]) or the later chill units [10] were simple, and they have been outperformed by the dynamic model chill portions [11]. The dynamic model was developed based on observations of peach trees (*Prunus persica*) under controlled conditions. It assumes that chill requirement is accumulated in buds by a two-step process. Initially, there is a reversible process of formation and destruction of a thermally labile precursor of chill accumulation. In a second step, when a critical amount of this precursor is accumulated, this gets permanently transferred as one portion of chill accumulation. This model aimed to represent some observations such as the range of effective temperatures, the negation of the chilling effect by high temperatures even when combined with low temperatures and the enhancing effect of altering moderate temperatures with chill temperatures. Although empirically it was quickly validated [12], the higher level of sophistication of this model was not fully justified by the current understanding of the physiological mechanisms controlling bud dormancy. At the physiological level, bud dormancy is controlled through the effect that chilling has on hormonal, metabolic, translocation and oxidative stress factors (Reviewed by Hernandez et al. [13]). Recent studies have contributed to understand that dormancy release in spring relies on hydrolysis of starch and mobilization of soluble carbohydrates, and trees have evolved to repress sugar mobilization during the winter warm spells to avoid the risk of frost damage [14]. This explains why trees require a stronger forcing stimulus (i.e. higher spring temperatures) when chill has been insufficient (i.e. high winter temperatures) [15].

The California Irrigation Management Information System (CIMIS) was developed in 1982 by the University of California, Davis with the main objective of assisting irrigators to estimate crop water needs and manage water resources more efficiently. This is achieved through the estimation of reference evapotranspiration (ETo) using the Penman-Monteith equation modified by Pruitt and Doorenbos [16]. This ETo is the estimation of the water consumption of a well watered reference crop, in this case grass with an approximate height of 3 inches, used in conjunction with a site and crop-specific crop coefficient (Kc) to estimate crop evapotranspiration (ETc). Weather stations (WS) designed for the estimation of ETo, have a regular maintenance schedule, but in addition these ETo stations need to be surrounded by grass which is well watered, fertilized and mowed regularly at a height of 3 inches. The continuous support of the CIMIS network has been crucial to increase crop water use efficiency in the California agriculture industry and to support hundreds of peer-reviewed irrigation studies that in turn have improved our understanding of irrigation management. This dataset has changed, adding and discontinuing some stations from the original 43 stations established across California in 1985 to the 151 stations online today. Over the years, it has accumulated long term datasets that have increased the interest of researchers out of the field of water resource management. Among the fields of study that benefited from CIMIS data are environmental ecology [17], plant biology [18], pests ecology [19] and local climate change projections [20].

**Fig 1:**
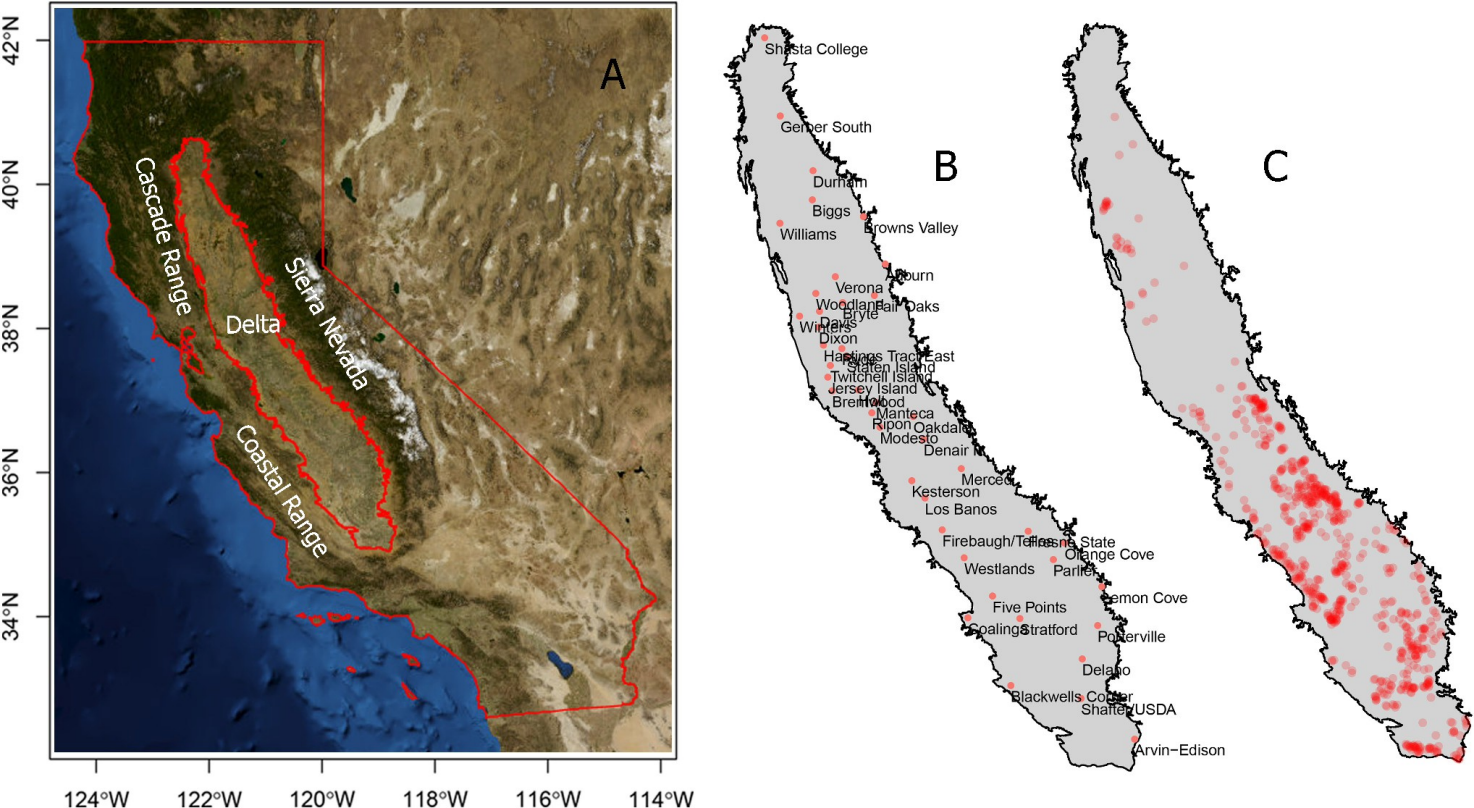
Overview of California’s central valley showing some landmarks such as mountain ranges and the Sacramento-San Joaquin river delta for reference purposes (USGS National Map Viewer; A), the location and name of the CIMIS stations (B) and the location of the in-orchard weather stations (C).

In the last decades we have experienced an increase and the accessibility of data at the service of agriculture industry and research. During the last years digital agriculture has acquired a new dimension and the central valley of California (**Fig 1A**) is a hotspot for this growth. Traditionally, it has been accepted to use the closest CIMIS WS to obtain weather records for research and commercial use. In this study we aimed to examine the potential differences between, temperature measurements, and temperature derived metrics GDD, and chill portions (CP), collected at in-orchard and CIMIS weather stations. In particular, we examined how interpolation error is reduced by increasing the number of stations and then we evaluate the discrepancies between actual measurements, localized interpolation, and the closest CIMIS station.

## 2. Materials and methods

### 2.1 CIMIS weather stations

CIMIS data from the 37 stations located in the central valley (**Fig 1B**) was downloaded and conditioned using the ‘ChillR R’ package [21]. This package fills gaps in temperature records interpolating the daily min and max and reconstructing hourly records using latitude as a proxy. Three stations that had more than 7% of missing data were discarded from the dataset. Given the habit of researchers and agriculture professionals of using the closest CIMIS stations data as the most representative of a site’s weather, Thiessen polygons were built to represent this using the ‘dismo’ package [22]. Thiessen polygons include any location that is closer to a given CIMIS station than any other CIMIS station.

### 2.2 In-orchard weather station network

The in-orchard weather dataset was provided by 731 stations commercially managed by Semios (S1 Fig) throughout the entire Central Valley but concentrated in few hotspots in the middle and south such as the corridor near the Southern Coastal Range, Merced, Fresno and the north east of Bakersfield (**Fig 1C**). The network consists of a proprietary in-orchard wireless mesh that transmits data over a cellular network to a cloud storage database for analysis. The mesh nodes are METER ATMOS 14 Gen 1 (METER Group, Pullman WA) sensors installed above the canopy and connected to agricultural sensor hubs which send information to the cloud database via gateways.

### 2.3 Calculation of thermal time and chilling

Thermal time was calculated from 10 min temperature records either using the daily (for GDD) or hourly (for GDH) mean temperature minus a base temperature (Tb) and truncating values above the maximum temperature (Tm)of each model. In brief, thermal time models account for the degrees above the Tb temperature up to the max temperature. Thermal time models can be optimized to represent the advance of different biological processes by adjusting Tb and Tm. The models for calculating thermal time used were: i) GDD with a Tb of 0°C ([23]; GDD_Tb0_), GDD with a Tb of 4.5°C ([24]; GDD_Tb4.5_), GDD with a Tb of 12.7°C and a Tm of 35°C ([25]; GDD_Tb12.7_Tm35_) and GDH with a Tb of 4.5°C and a Tm of 25°C ([10]; GDH_Tb4.5_Tm25_). To enable the comparison with the literature these metrics were calculated for the 3 quarters (S1 File). To simplify, we took GDD_Tb0_ as the reference for showing data in figures. GDD and GDH were summed for the first, second and third quarter of 2020 (Q1, from the 1st of January to the 31st of March; Q2, from the 1st of April to the 30th of June; and Q3, from the 1st of July to the 31st of September). Chill portions were calculated as described by [11] using hourly records from the 1st of September till the 28th of February (Chill period).

### 2.4 Crop and pest phenology models

For assessing the relevance of accurate site-specific weather data we calculated the predictions of crop and pest phenology models. Grapevine bud break and veraison dates were calculated using the thresholds of 1217 and 2547 GDD_Tb0_, respectively, proposed by Parker et al. [2] for Chardonnay variety. We estimated almond flowering dates assuming a sequential completion of chill (23CP) and heat requirements (300GDD_Tb4.5_) [7] which is a simplification of the tradeoff between chill and heat accumulation [26]. As an example of modeling pest phenology we followed the assumptions of Patak et al. [4] of a heat requirement of 148 GDD_Tb12.7_Tm35_ for NOW to lay eggs, 565 GDD_Tb12.7_Tm35_ for the first generation developing from mummy nuts, and 444 GDD_Tb12.7_Tm35_ for any subsequent generation in almonds and walnuts [27]. This simplification of the NOW lifecycle is useful for examining these data sets at scale. However, we recognize that second generation NOW may also develop on mummy nuts if new crop nuts are not available and therefore models intended to inform farm-level management could likely be improved by accounting for factors.

### 2.5 Spatial interpolation

**Fig 2:**
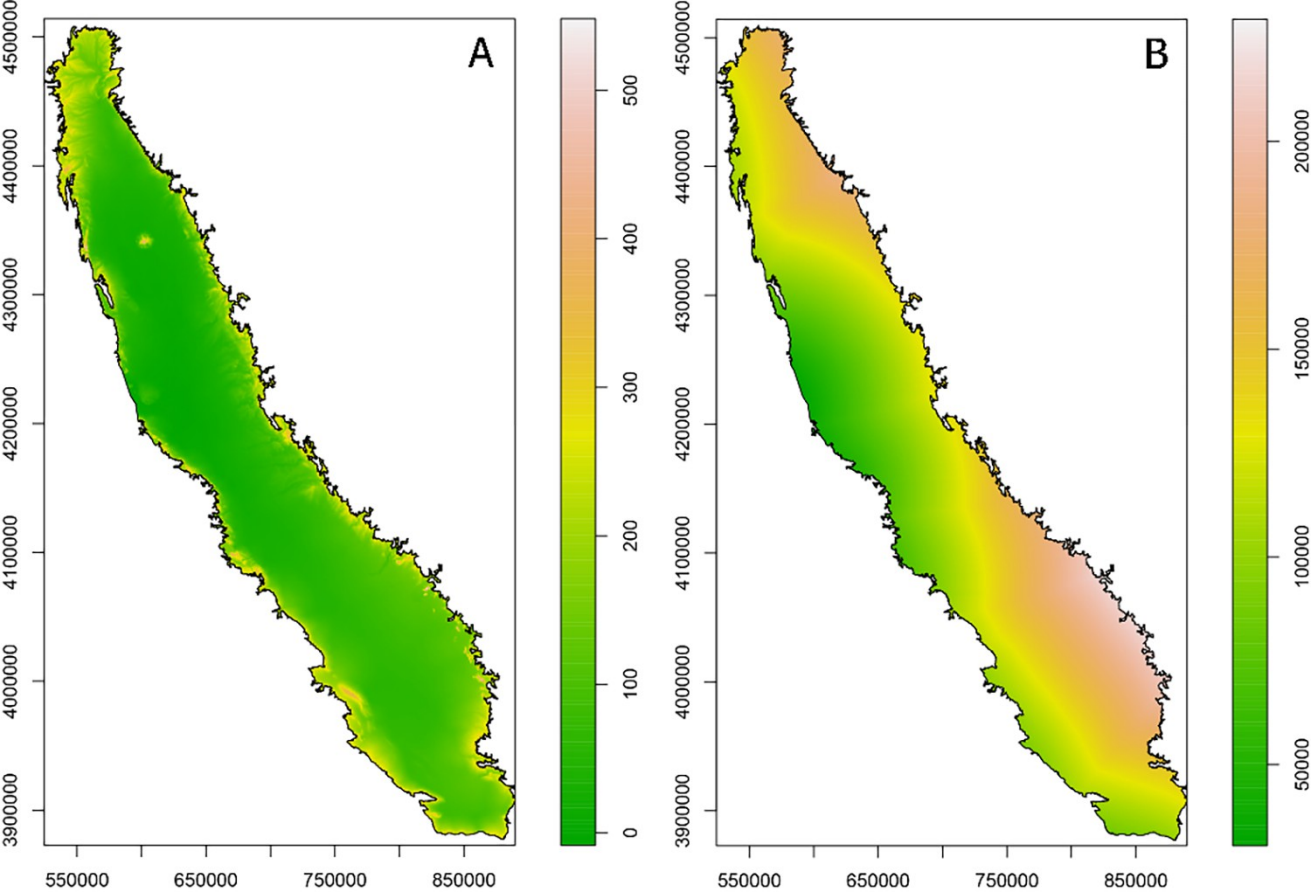
Flat projection (WGS84 zone 10) of auxiliary variables elevation in meters (A) and distance to the coast in meters (B) used for kriging thermal time, chill accumulation and phenology variables.

Kriging techniques predict values at unsampled locations using a spatial model that considers the distance between observations. Kriging interpolation was performed using the *automap* R package [28] supervising the automatic fit of the variogram model in each case (S2 File). The variogram model is a function of semivariance against distance. A Gauss-Newton fitting for variograms was performed with automated initial values for nugget, range and sill following the methods in Hiemstra et al. [28]. This technique takes into account trends in the data across auxiliary variables (co-kriging), in our case elevation and proximity to the coast. Co-kriging is typically used when the target variable (i.e. weather) is expensive and in turn we have high resolution grid of points for another variable (i.e. elevation and distance to the coast) that may share a similar spatial structure. The latitude and longitude of the CIMIS and in-orchard stations was reprojected onto flat coordinates (WGS84, zone 10). Digital elevation model of California (**Fig 2A**) with a resolution of 90m was reprojected into flat coordinates and resampled for the final resolution of 1km. Distance to the coast was calculated as the minimum distance to the continental shoreline of the USA with a final resolution of 1km (**Fig 2B**). For detail step by step guide see the code shared.

### 2.6 Cross validation

In order to test the marginal performance gain of additional stations to the CIMIS network we sampled randomly an incremental number of stations (15, 30, 60, 100, 150, 250, 350, 450, 550, 650) 10 times among the 731 additional stations and then performed ten 10-fold cross validations for mean temperatures and GDD during the Q1 (1 January to 31 March 2020), Q2 (1 April to 30 June 2020) and Q3 (1 July to 30 September 2020) of 2020 and the mean temperatures and chill portions during the chill period (1 September 2020 to 28 February 2021).

## 3. Results

### 3.1 Spatial variability of GDD across the Central Valley

**Fig 3:**
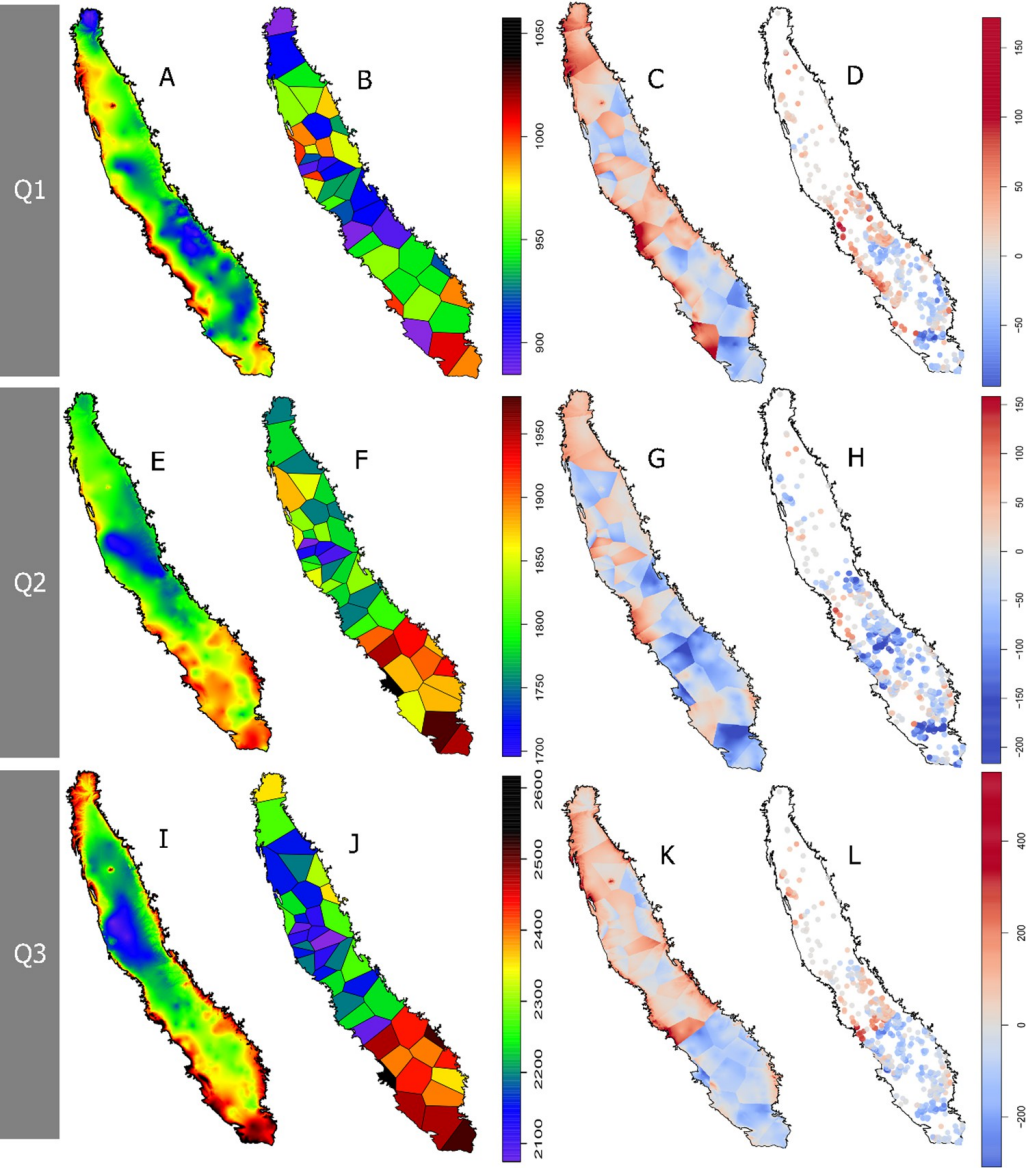
GDD_Tb0_ accumulation interpolated using CIMIS and 731 in-orchard stations (A,E,I), GDD_Tb0_ accumulation for the closest CIMIS station (B,F,J), discrepancy between interpolation and closest CIMIS (in-orchard/CIMIS interpolation minus closest CIMIS value; C,G,K) and discrepancy between each of the 731 in-orchard stations and their closest CIMIS station (in-orchard minus CIMIS value) (DHL) during the first (Q1; A,B,C,D), second (Q2; E,F,G,H) and third (Q3, I, J, K, L) quarter of 2020.

Although the Central Valley is a great plain only interrupted by the Sutter Buttes, there was a great difference in GDD accumulation within short distances (**Fig 3A, 3E and 3I**). In Q1 (**Fig 3A**), some of the coldest points (900GDD) were concentrated along the belt from Sacramento to Fresno. However, a few dozens of miles to the west near the Southern Coast Range, we found some of the warmest points (1000GDD). In Q2 (**Fig 3E**) and Q3 (**Fig 3I**), we found the cooler spots in the delta and the warmest regions were more concentrated in the southern part of the Central Valley.

Assigning the value of the closest CIMIS to each pixel (Thiessen polygons; **Fig 3B, 3F and 3J**) revealed strong shifts depending what station was closest. For instance, in Q1 (**Fig 3B**), some of the highest and lowest values were found in neighbouring CIMIS stations, Shafter (1011.8 GDD) and Blackwells Corner (870.6 GDD). In Q2 (**Fig 3F**), the most discrepant neighbours were Coalinga (2027.6 GDD) and Blackwells Corner (1866.4 GDD), whereas in Q3 (**Fig 3J**), these were Westlands (2480.2 GDD) and Firebaugh (2077.9 GDD).

When comparing interpolated values with the closest CIMIS station (i.e. GDD from Fig 3A, 3E and 3C interpolations minus GDD from Fig 3C, 3G and 3K interpolation, respectively) (**Fig 3C, 3G and 3K**), discrepancies of different magnitudes were found depending on the quarter and station. In the overall, CIMIS stations in the south accumulated more GDD than the in-orchard stations interpolation, up to 150 GDD in Q1 (**Fig 3C**), 150 GDD in Q2 (**Fig 3G**) and 400 GDD in Q3 (**Fig 3K**). In the comparison between GDD accumulated at each of the in-orchard stations with their closest CIMIS (**Fig 3D, 3H and 3L**), the difference between these two (i.e. GDD accumulated at each in-orchard station minus GDD accumulated at their closest CIMIS station) showed great discrepancies. For instance, in Q1 (**Fig 3D**), WS located in commercial orchards accumulated ca. 50 GDD more than their closest CIMIS through most of the corridor near the Southern Coast Range. However, some of these orchards near the Coast Range accumulated nearly 150 GDD less during Q2 (**Fig 3H**) and Q3 (**Fig 3L**). Orchards in the Bakersfield area accumulated consistently less GDD throughout Q1 (50 GDD), Q2 (200GDD) and Q3 (200GDD) than their respective closest CIMIS station.

### 3.2 Spatial variability of CP across the Central Valley

**Fig 4:**
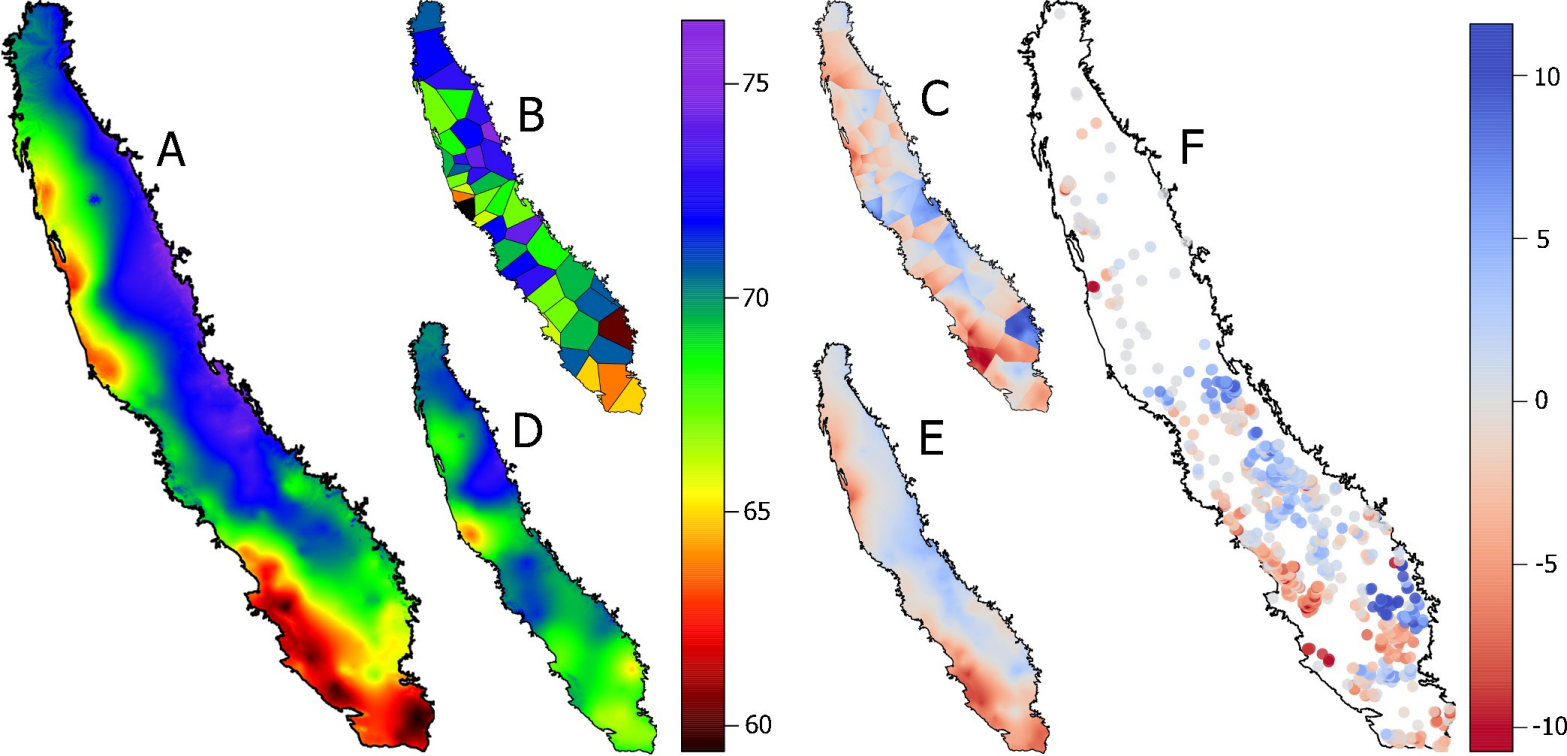
Chill accumulation (chill portions) interpolated using CIMIS and 731 in-orchard stations (A), GDD_Tb0_ accumulation for the closest CIMIS station (B), discrepancy between interpolation and closest CIMIS (C), chill accumulation interpolated using only CIMIS stations (D), discrepancies between interpolations using CIMIS and in-orchard stations combined and CIMIS stations only (in-orchard/CIMIS minus CIMIS value; E) and discrepancy between each of the 731 in-orchard stations and their closest CIMIS station (in-orchard/CIMIS interpolation minus closest CIMIS value; F) during the chill period (from September 1st 2020 to February 28th 2021).

Chill accumulation map generated by combining both in-orchard and the CIMIS datasets (**Fig 4A**) showed clear spatial trends where most locations close to the Coast Ranges and the South accumulated much less CP than the rest. The highest chill accumulation recorded in both in-orchard and CIMIS weather stations were 78CP near the Northern part of Sierra Nevada (S1 Data; **Fig 4B**). Contrary, 43 of the in-orchard stations recorded a chill accumulation below 60CP, while the lowest CP recorded by the CIMIS station was 59 CP at Porterville and Brentwood (S1 Data; **Fig 4B**). These two CIMIS stations were surrounded by other CIMIS stations that in some cases showed major shifts in chill accumulation. Porterville with an elevation of 121m accumulated 59.3 CP while Lemon Cove just 32 km away with an elevation of 151m had 73.8CP. The kriging model using only CIMIS stations showed medium values for that area (**Fig 4D**). Some of the greatest differences between the krigged CP map using all stations and just CIMIS were actually observed in the Porterville area (**Fig 4C**). Therefore, using the closest CIMIS area we would have obtained 10 CP less than the interpolation using all data available. Other major discrepancies were also found in the Southwest of the Central Valley where none of the CIMIS stations recorded below 60 CP (**Fig 4B**). In fact, Blackwells Corner recorded 71CP while the in-orchard stations within less than 20km recorded between 57 and 60.6 CP. Not surprisingly, when kriging chill accumulation only using CIMIS stations(**Fig 4D**) the South West of the Central Valley did not show such low chill accumulation (**Fig 4A**) and the discrepancies between the kriging of both data sets were greatest (**Fig 4E**). When comparing individual in-orchard stations to their closest CIMIS station (**Fig 4F**), differences in chill accumulation were clearly clustered, suggesting that the difference between in-orchard and CIMIS stations was greater than the differences among in-orchard stations.

### 3.3 Discrepancies in crop and pest phenology predictions between in-orchard and CIMIS stations

There were significant discrepancies between the model forecasts when the closests CIMIS station was used as an equivalent to the in-orchard station (**Table 1**). Grape phenology models showed the smallest discrepancies, with RMSE of 2.93 and 3.29 days for flowering and veraison, respectively. However, some stations showed discrepancies as high as 10 and 12 days for chardonay flowering and veraison, respectively. In the case of almond phenology, RMSE was 8.3 and 6.29 days for almond flowering and hulsplit, respectively. Furthermore, the largest differences were found to be up to a whole month off in the case of almond flowering and 19 days for hullsplit. The RMSE for the number of NOW generations was 0.48, which is more than a 10% deviation from the average 4.02 generations forecasted for the area of study.

**Table 1:** Minimum, mean, maximum and RMSE of the discrepancies between in-orchard stations and their closest CIMIS.

			in orchard—closest CIMIS station difference			
Variable	Units	Model source	min	mean	max	RMSE
Distance	meters		440	18611	49860	
Chardonnay flowering	days	Parker et al., [2]	-4.00	1.97	10.00	2.93
Chardonnay veraison	days	Parker et al., [2]	-12.00	-0.90	8.00	3.29
Almond flowering	days	Parker and Abatzoglu [7]	-13.00	2.73	30.00	8.30
Almond hullsplit	days	Tombessi et al., [35]	-19.00	-3.84	7.00	6.29
NOW	generations	Patak et al., [4]	-0.81	0.23	1.58	0.48

### 3.4 Spatial variability of crop and pest phenology

**Fig 5:**
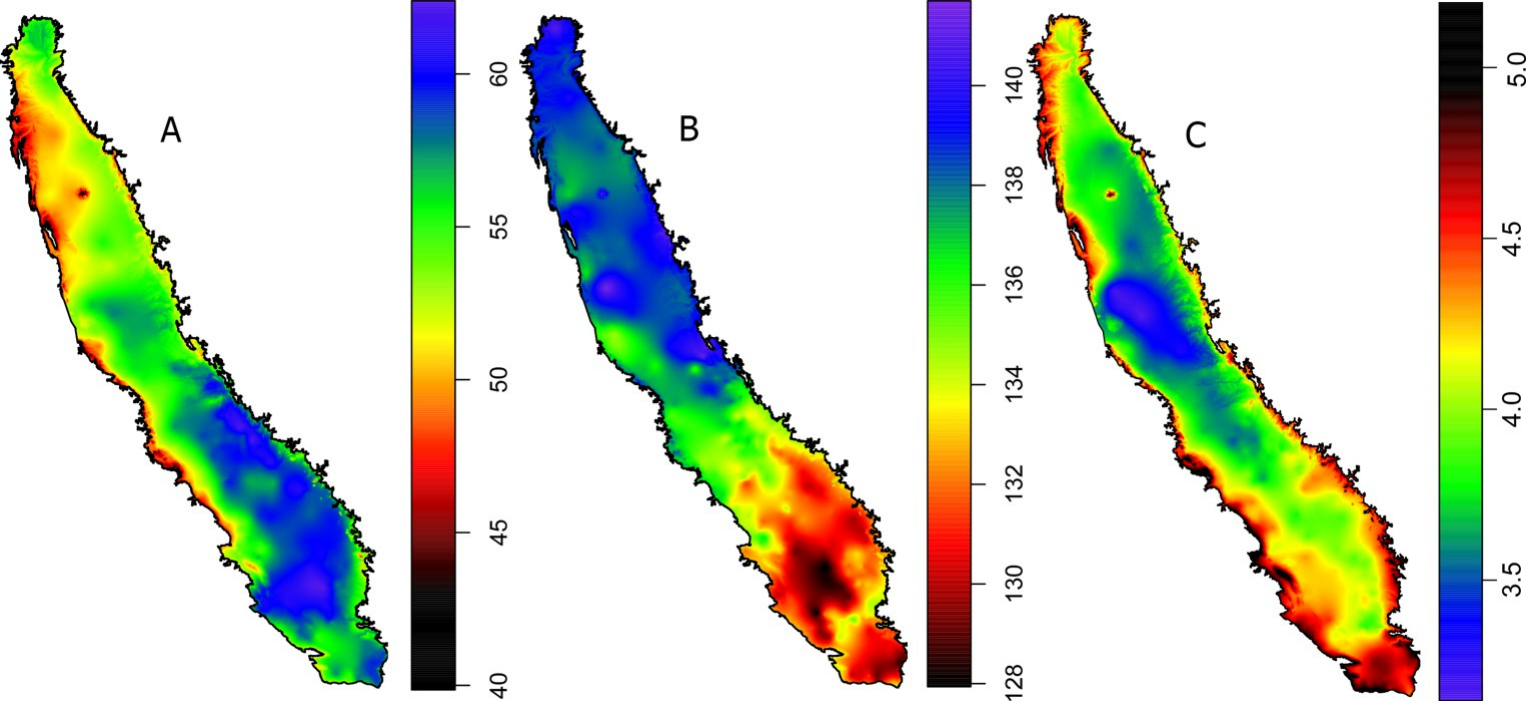
Interpolation of Almond full bloom prediction after the sequential completion of chill requirement (23CP) and heat requirement (300 GDD_Tb4.5_) (A), 1% Hullsplit prediction after using the GDD_Tb5_Tm35_ during the 90 days after flowering in a linear regression model proposed by Tombessi et al. [35] (B), and number of NOW generations predicted using the accumulation of GDD_Tb12.7_Tm35_ [4] (C) for season 2020 using data from CIMIS and 731 in-orchard stations.

The spatial pattern of almond flowering predictions showed earliest dates in the northwest side of the Central valley due to the faster accumulation of 23 CP in that region (**Fig 5A**). This pattern was not transferred to hull split predictions where there was a clear South-North gradient across the Central Valley (**Fig 5B**). The spatial pattern of the NOW generations predicted by GDD_Tb12.7_Tm35_ accumulation had also a strong South-North gradient where the locations near Bakersfield, the Southern Coast Range and the South of the Sierra had potentially up to 5 NOW generations (**Fig 5C**).

**Fig 6:**
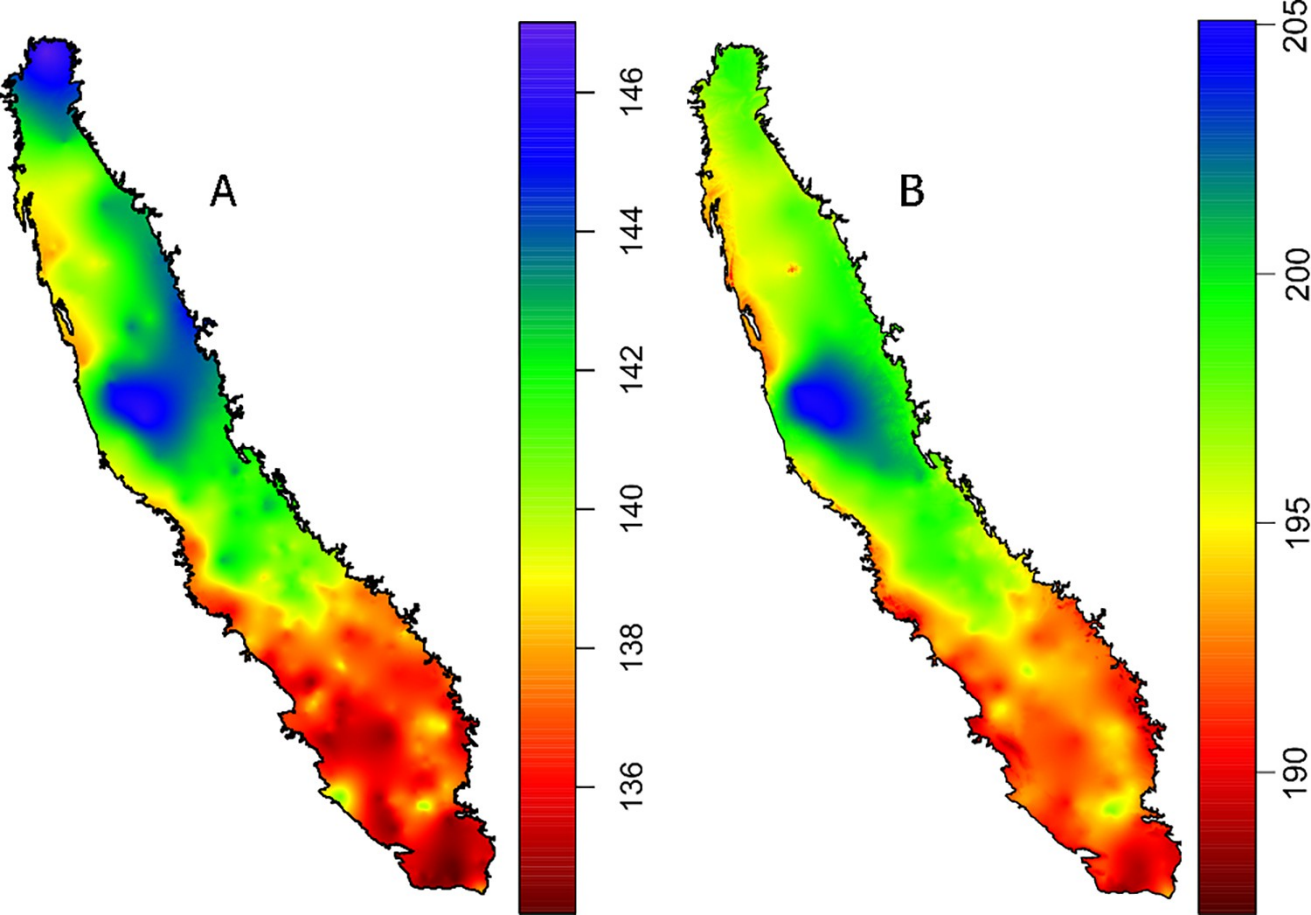
Interpolation of Grape (cv. Chardonay) 50% flowering prediction assuming a heat requirement of 1217 GDD_Tb0_ after julian day 60 (A) and 50% Veraison prediction assuming a heat requirement of 2547 GDD_Tb0_ after julian day 60 using data from CIMIS and 731 in-orchard stations.

Grape phenology predictions showed that the southernmost third of Central Valley had an earlier flowering (ca. 136 doy) and veraison (ca. 190 doy), compared to the rest of the Central Valley but especially in the area of the delta where flowering was predicted for doy 146 and verison for doy 204 (**Fig 6**).

**Fig 7:**
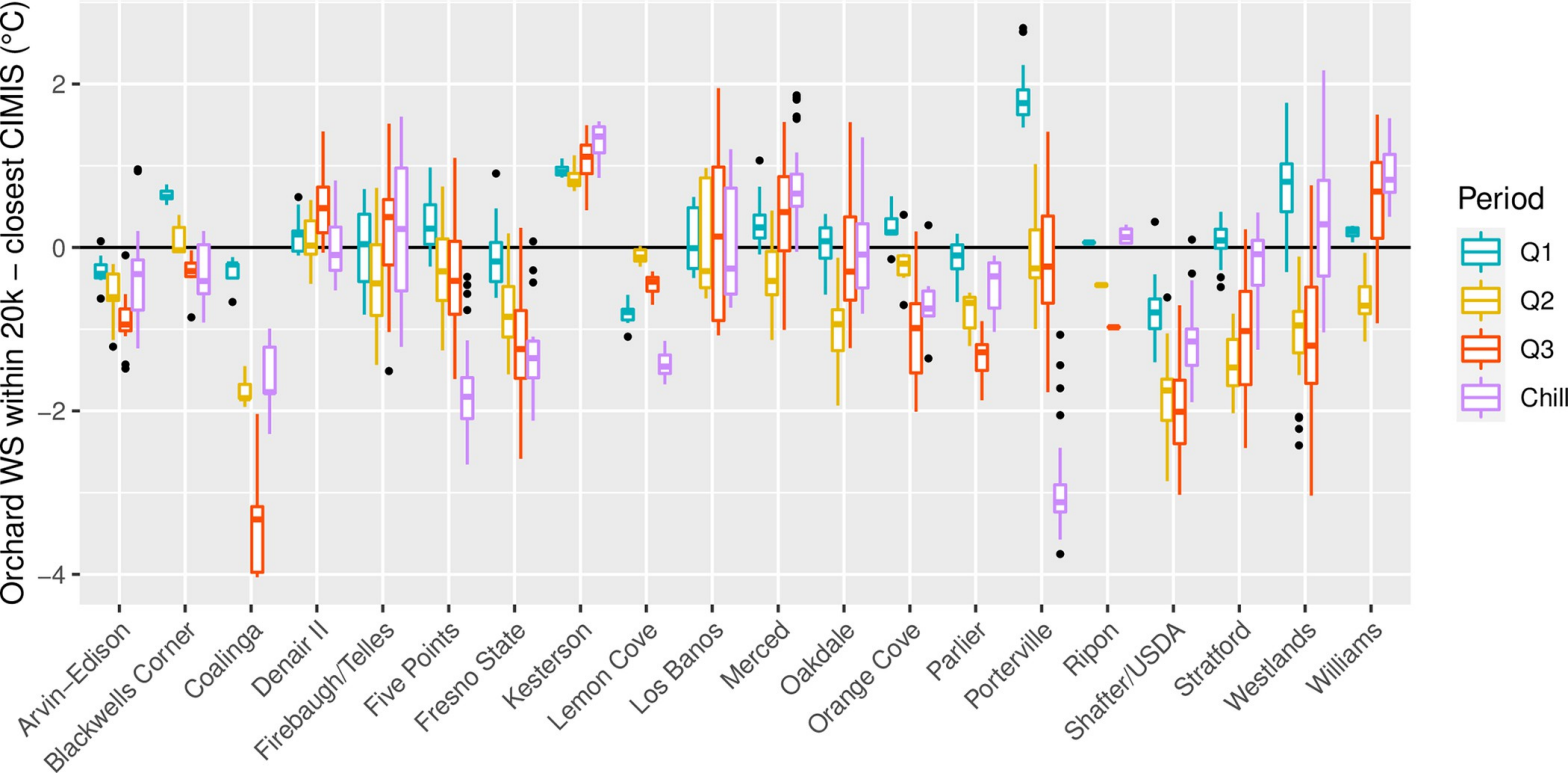
Discrepancies between in-orchard stations within 20 kilometers from a CIMIS station and that CIMIS station mean temperature during the first (Q1), second (Q2), and third (Q3) quarter of 2020, and the following chill period (chill; September 1st 2020 to February 28th 2021).

When comparing the mean temperatures of each CIMIS station to the in-orchard WS located less than 20km some strong discrepancies also arise (**Fig 7**). The greatest discrepancy was found for Coalinga station where during Q3 it was on average 3.3°C degrees higher than the average of the surrounding in-orchard WS, and went down to 1.8°C difference for Q2 and chill period. Porterville temperatures started the year 1.8°C lower than the orchards (Q1), became similar with a great variability for Q2 and Q3, and ended with 3.2°C higher during the chill period. Some other stations to be highlighted are Fresno State, Shafter, that had consistently higher temperatures, or Kesterson that had consistently lower temperatures than the surrounding orchards.

The closest in-orchard WS to a CIMIS station was located 440 m from Denair II and had deviations of 0.17, -0.04, 0.12 and -0.09°C (Q1, Q2, Q3 and chill period). The second closest in-orchard station was 1260 m away from Merced with deviations of -0.01, -0.44, 0.84 and 0.61°C (Q1, Q2, Q3 and chill period). On average, in-orchard WS were 0.11°C higher in Q1, 0.68°C lower in Q2, 0.57°C lower in Q3 and 0.60°C lower than CIMIS stations during the chill period (S1 Data).

### 3.5 The error of kriging was reduced by additional stations

We examined the effect of additional stations to kriging predictions through the coefficient of determination (R^2^) and the root mean squared error (RMSE) of a series of cross validations (**Table 2**). Initial R^2^ for using the CIMIS network came very low for all the variables tested (<0.20) but one, Q3 mean temperature (0.69) where the dependent variable happened to be tightly correlated to latitude (0.37) and longitude (0.54). Those R^2^ increased as orchard stations were added to the kriging but at different rates. While most of the variables reached a R^2^ above 0.6, some of them took only 30 additional stations to reach 0.3 whereas others took 60 or even 100 more stations to reach that threshold. RMSE of temperatures also showed great improvement as we added stations reducing to less than half the error of using CIMIS only. Similar reductions of error were observed for GDD and CP accumulation where in most cases RMSE dropped down to half. The case of the chill accumulation is remarkable whereas the R^2^ went up from 0.09 to 0.74 while the RMSE just decreased by 35%.

**Table 2:** Coefficient of determination (R^2^) and root mean square error (RMSE) of the cross validation of kriging adding an incremental number of in-orchard stations (15,30,60,100,150,250,350,450,550 and 650) to a CIMIS baseline (0).

	Q1								Q2								Q3								Chill period							
	Mean temperature				GDD				Mean temperature				GDD				Mean temperature				GDD				Mean temperature				CP			
Stations added	R^2^		RMSE		R^2^		RMSE		R^2^		RMSE		R^2^		RMSE		R^2^		RMSE		R^2^		RMSE		R^2^		RMSE		R^2^		RMSE	
0	0.01	±0.02	0.55	±0.01	0.03	±0.08	41.6	±1.9	0.16	±0.03	0.89	±0.02	0.18	±0.03	74.6	±2.2	0.70	±0.02	0.79	±0.01	0.63	±0.02	89.7	±6.4	0.12	±0.05	0.63	±0.02	0.09	±0.06	3.71	±0.17
15	0.09	±0.04	0.50	±0.03	0.07	±0.05	37.1	±1.7	0.27	±0.05	0.75	±0.04	0.29	±0.07	66.0	±5.6	0.66	±0.04	0.93	±0.10	0.65	±0.04	94.1	±10.3	0.16	±0.06	0.57	±0.02	0.29	±0.09	3.47	±0.28
30	0.14	±0.06	0.46	±0.02	0.17	±0.09	36.2	±1.1	0.23	±0.09	0.75	±0.05	0.37	±0.10	60.2	±4.2	0.68	±0.06	0.88	±0.05	0.68	±0.04	86.2	±9.0	0.25	±0.05	0.52	±0.02	0.34	±0.17	3.61	±0.45
60	0.22	±0.07	0.42	±0.02	0.24	±0.08	32.6	±1.7	0.38	±0.08	0.63	±0.03	0.47	±0.10	52.6	±3.9	0.66	±0.09	0.82	±0.09	0.62	±0.06	83.0	±4.9	0.30	±0.09	0.47	±0.02	0.48	±0.15	3.23	±0.44
100	0.33	±0.05	0.37	±0.01	0.37	±0.06	28.1	±1.9	0.49	±0.04	0.56	±0.02	0.57	±0.06	45.8	±2.4	0.62	±0.03	0.78	±0.04	0.62	±0.05	74.2	±5.5	0.41	±0.04	0.42	±0.01	0.53	±0.14	3.11	±0.43
150	0.39	±0.06	0.34	±0.02	0.43	±0.06	27.4	±1.6	0.56	±0.06	0.50	±0.03	0.57	±0.06	43.6	±2.0	0.63	±0.05	0.72	±0.03	0.61	±0.06	69.0	±3.2	0.46	±0.05	0.39	±0.02	0.61	±0.10	2.97	±0.36
250	0.53	±0.04	0.29	±0.01	0.51	±0.05	24.2	±1.1	0.61	±0.02	0.46	±0.01	0.63	±0.04	40.1	±1.8	0.61	±0.05	0.68	±0.04	0.62	±0.03	63.0	±1.7	0.52	±0.05	0.35	±0.02	0.68	±0.02	2.63	±0.16
350	0.57	±0.04	0.27	±0.01	0.58	±0.03	22.5	±1.0	0.65	±0.04	0.41	±0.02	0.67	±0.03	36.9	±1.4	0.64	±0.02	0.63	±0.02	0.65	±0.02	59.1	±1.8	0.60	±0.04	0.31	±0.01	0.70	±0.02	2.55	±0.10
450	0.64	±0.02	0.25	±0.01	0.61	±0.04	21.4	±0.8	0.68	±0.02	0.40	±0.01	0.69	±0.02	35.5	±1.2	0.66	±0.01	0.60	±0.01	0.63	±0.03	57.2	±1.8	0.62	±0.02	0.30	±0.01	0.72	±0.02	2.50	±0.10
550	0.66	±0.02	0.24	±0.01	0.65	±0.01	20.5	±0.5	0.71	±0.02	0.38	±0.01	0.71	±0.01	34.1	±0.5	0.66	±0.02	0.58	±0.01	0.66	±0.02	55.1	±1.3	0.65	±0.02	0.28	±0.01	0.73	±0.01	2.44	±0.04
650	0.68	±0.01	0.23	±0.00	0.67	±0.01	19.94	±0.3	0.73	±0.01	0.36	±0.01	0.74	±0.01	32.35	±0.5	0.67	±0.01	0.57	±0.01	0.66	±0.01	54.07	±0.48	0.66	±0.01	0.27	±0.01	0.74	±0.01	2.40	±0.04

## 4. Discussion

### 4.1 Using a greater resolution revealed great spatio-temporal variability

In times of the explosion of digital agriculture, weather related parameters are still the main driver of food production, and accurate weather data is key to calibrate and operate the great majority of agricultural models. For instance, weather and its derived parameters are a great component of integrated pest management models [29], crop phenology [23], and yield estimation [30]. Just like California’s permanent crop industry uses CIMIS, there are a lot of studies that used CIMIS or other relatively low resolution datasets in the Central Valley. Luedeling et al. [20] was a pioneer study that used CIMIS and National Climatic Data Center (NOAA) historical data combined with climate change projections to make localized estimations of chill accumulation by the end of the century. In a recent study, Pathak et al. [4] studied how many generations of NOW could occur in a season under 10 different climate change projections. However, the greatest resolution of these projections was 1° by 1° (111 km). Those and similar studies contribute to greatly improving the understanding of how temperatures, and all the processes that depend on them, will evolve through the next decades at the county or state level. However, the present study provides evidence that the weather spatial variability is much greater than what we initially expected, and the Central Valley has a great range of mesoclimates sustained not only by latitude and elevation, but also by thermal inversion and openings to the sea and the mountain ranges [31]. To our knowledge, this is the first time an analysis of such a high density of weather stations has been reported over such a great area.

### 4.2 The discrepancies between CIMIS and in-orchard stations were largely due to mesoclimate variability

Discrepancies between CIMIS weather stations and in-orchard stations were expected. In the first place, CIMIS, as any other reliable weather station network, aims to capture air temperatures unbiased by factors such as surrounding trees or the irradiation from bare soils. Whereas in-orchard stations aim to capture air temperatures affected by all the conditionings that also affect the trees and pests living there. It is noteworthy that the stations we used were inside the orchards above the canopy. However, temperatures inside the canopy can be on average 1.2°C lower for the minimum and 2°C higher for the daily maximum temperature [32]. There is also the distance factor, as this study did not count with a side by side comparison of a CIMIS station with an in-orchard station on the same site. A good proportion of the in-orchard stations located within 2 km of a CIMIS station had discrepancies, and in the overall in-orchard stations were 0.4°C cooler on average for the 14 months of data used. However, the in-orchard stations within 20km of a CIMIS station had greater discrepancies between them than with their closest CIMIS station, suggesting that most of these discrepancies were due to actual shifts in mesoclimates within those small distances.

In this study, we showed how the Central Valley, one of the most well studied agricultural hotspots in the world, could benefit from on-site weather records. CIMIS is a research-grade and high-maintenance network of WS, mainly due to the need of pyranometers (among other sensors) and a reference crop maintenance. While these are necessary for an accurate estimation of reference evapotranspiration, it can be a limitation for obtaining a high resolution network of temperature records. One limitation is the cost and maintenance but also the need of a public site or collaborator to install the devices. In addition, temperature records from weather stations may not always be representative of the agroecosystem microclimates. Orchards have a seasonal pattern in vegetation cover where trees are leafless and on bareground during fall. With the first rains of the winter, cover crops start to grow, in spring, trees leaf out while cover crops gradually dry off. The presence of vegetation and irrigation can have an effect on relative humidity and air temperature through evaporative cooling [33]. Therefore, WS located in an orchard may have a seasonal bias with respect to air temperature (typically measured over a well-maintained lawn) [16].

### 4.3 Implications of thermal time discrepancies for crop decision-making

The discrepancies shown in this study ranged from 50 GDD in Q1 to over 300GDD during the Q3. During most of Q1, trees do not have leaves although they are quite sensitive to GDD and phenomena such as budbreak and flowering can be advanced significantly. For almonds, which tend to flower in the Central Valley by the end of February they have a heat requirement of 5300–8900 GDH_Tb4.5_Tm25_ for almonds in general and 6800 GDH_Tb4.5_Tm25_ (equivalent to 620 GDD_Tb0_) for Nonpareil in particular (Rattigan and Hill, 1986). Given that the equivalence of those 6800 GDH_Tb4.5_Tm25_ to 620 GDD_Tb0_ within our Q1 dataset (S1 File), and that the average daily accumulation of GDD_Tb0_ was 10.4, a discrepancy from 150 to -75 GDD_Tb0_ can results in an error of up to two weeks for flowering predictions. Other species flower later and have a higher heat requirement. For instance, heat requirement for pistachio varieties in California is estimated to be 11500 GDH_Tb4.5_Tm25_ [34] which could increase the error in the budbreak forecast. The spatial pattern in almond flowering prediction for year 2020 in this study was very similar for the average conditions through the last decades reported by Parker and Abatzoglu [7] with the same methods, where the latest flowering occurred in the central South part of the Central Valley. This finding suggests that the spatial pattern observed in 2020 is representative of the average conditions during longer periods.

The 90 days immediately after almond flowering (typically from the end of Q1 to the end of Q2) is the period when thermal time is accounted to predict 1% hull split [35]. This phase is one of the key milestones in the growing season for almonds as this is the point when the nuts become susceptible to navel orangeworm (NOW; *Amyelois transitella*) and when regulated deficit irrigation is applied to promote nut development. The discrepancies found between CIMIS and in-orchard station of up to 175 GDD_Tb0_ observed in some stations in Q2 accounts for 19.5% (assuming equivalence) of the 900 GDD_Tb5_Tm35_ accumulated on average during the 90 days after flowering in Tombesi et al., [35]. In that study, a discrepancy of 175 GDD_Tb0_ would be equivalent to a shift in hull split date of 10 days. The spatial pattern of our hull split prediction for year 2019 had a marked gradient where the South showed the earliest dates and progressively increased for higher latitudes. The spatial trends shown in our results again were the same as for the average present conditions and climate change predictions throughout the 21st century reported by Parker and Abatzoglu [7].

### 4.4 Implications of chill accumulation discrepancies for crop decision-making

The level of completion for chill requirement is an important feature in the prediction of flowering date [36]. However, for Pistachios in California there is an actual chance of not meeting chill requirements of certain varieties such as Kerman which could be as high as 69 CP, but yields only being affected by chill accumulation lower than 57 CP [37]. Among the effects of lack of chill are: delayed budbreak, blind lateral buds, asynchrony with pollinator cultivar, and poor fruit set leading to lower yields [38]. During the pistachio chill period of season 2021, significant areas of the Central Valley accumulated 60 CP or less mainly in the Southwest of the Central Valley and some areas near the Northern Coast Range. This was only noticeable when in-orchard stations were used, and in fact a large number of in-orchard stations near the Northern Coast Range recorded from 5 to 12 CP less than their closest CIMIS station. This was likely related to the poor representativity of some CIMIS stations such as Coalinga and Blackwells Corner, which are very influenced by their proximity to an opening to the coast. Although in most cases lack of chill is only noticed when symptoms occur, having an accurate monitorization of the level of completion of chill requirement can be important for proactive management. In fact, poor chill accumulation can be palliated by the application of hormone homologs, calcium ammonium nitrate and hydrogen cyanide when a certain fraction of the chilling requirement has been met [39].

### 4.5 Implications of crop phenology prediction discrepancies for crop decision-making

During the second quarter of the year, grapes transition from bud break to flowering/fruit set and from fruitset to *veraison*. It is known that the length of the period from bud break to flowering has a higher responsiveness to increasing temperatures than the period from fruitset to *veraison* [40]. The difference between the earliest and the latest *veraison* forecast was 16 days; however, a similar spatial pattern was already present by flowering with a 12 day range of measurements. The varieties and wine styles grown in a region are strongly linked to thermal time accumulation and timing of phenological events. In most viticultural regions, it is assumed that the most suitable grape variety and growing system is one that matures towards the end of the growing season, avoiding the warmest part of the year during ripening. In California’s Central Valley, high yields are successfully grown harvesting weeks or months before the end of the growing season [41]. This study showed a forecast for Chardonnay’s *veraison* starting in the second week of July, which would mean a most certain harvest taking place before mid August.

The discrepancies between orchard stations and their nearest CIMIS station were high enough to affect the potential suitability of a crop or variety. For instance, thermal time accumulation during Q3 showed the greatest discrepancies (ca. 300 GDD_Tb0_). Winkler scale [42] classifies wine regions and their potential wine varieties, style and cropping system according to their GDD_Tb10_. The ranges of values within these Winkler scale regions are between 278 for Regions II, III and IV) to 478 GDD wide for Region V. Although most of the Central Valley falls into region V, the discrepancies observed in Q2 and Q3 could lead to some changes in the distribution of region 4 and 5 across the Central Valley reported by Jones et al. [43].

### 4.6 Implications of pest phenology prediction discrepancies for crop decision-making

The control of grape berry moth (*Paralobesia viteana*) relies on accounting for 700 GDD_Tb8.4_ (Tb = 8.4°C) from the 1st of March before applying a selective pesticide [44]. However, with such discrepancies in GDD_Tb0_ accumulation across Q2 there is a likely loss of efficiency in the application of pesticides. For nut tree crops, NOW is the most damaging pest in California [45]. The control of NOW relies on the application of insecticides and/or mating disruption within an integrated pest management program. Implementation of these tactics are most effective when timed to correspond with pest and host specific life-stages. For example, insecticides for NOW management are typically applied at hullsplit, which is when almonds become susceptible to NOW damage [8, 46]. However, our analysis showed that phenology predictions using the closest CIMIS station would add a RMSE of 8.3 and 6.29 days to flowering and hullsplit predictions, respectively, which would complicate the timing of these important NOW sprays. Furthermore, our data suggests 10% increase in the number of NOW generations per year. These high-level population trends could ultimately influence the timing of orchard sanitation practices, as well as the timing and duration of mating disruption deployment.

According to Patak et al. [4], NOW has up to four generations in counties like Kern and Kings, whereas three generations is far more common in the rest of the Central Valley. Following the same methods, our data showed great spatial variability. Areas close to the Coast Ranges and the southern Sierra Nevada had enough GDD_Tb12.7_Tm35_ for more than four generations while in the South of Bakersfield GDD_Tb12.7_Tm35_ accumulation was enough for five generations. In addition, our analysis used the most conservative parameters as the heat requirement for NOW developing on new crop nuts which was 444 GDD_Tb12.7_Tm35_ for almonds compared to the 402 GDD_Tb12.7_Tm35_ required for pistachios [27, 47]. This suggests that in our current conditions we may not be so far from having five generations of NOW within a season as initially proposed by Pathak et al. [4]. When considered at the level of individual farms or management zones, differences between in degree day accumulations ending Q3 was 300 GGD_Tb0_, and during this period the average daily accumulations in Q3 was 25 GGD_Tb0_. Therefore NOW model prediction for Q3 could differ between data sources by as much as 12 calendar days.

## 5. Conclusions

Although the Central Valley of California is mostly flat, this study showed a strong gradient across it but also some strong localized gradients related to the proximity to mountain ranges or openings to the ocean. We found some hot spots where thermal time was higher, leading to more NOW generations than what other studies reported using lower resolution weather data. When comparing the effectiveness of a interpolated continuous layer using only CIMIS stations, the RMSE of the mean temperature ranged from 0.55 to 0.89°C across all the periods studied. This error translated into 41–89 GDD per quarter or 3.7 CP across the chill period. These errors decreased by adding more stations but plateau after adding 250 weather stations to the kriging dataset, suggesting that the marginal contribution of additional stations with the same spatial distribution was smaller. This suggests that kriging weather data may be the most cost effective solution after a critical number of stations has been reached. When comparing in-orchard weather stations to their closest CIMIS station the RMSE was up to 8.3 days for some phenological stages which may be added to the error of the models itself. This shift in predictions is enough to create significant reductions in the efficiencies of practices such as pesticide application or mating disruption. Given the discrepancies the between nearest regional weather station and the weather stations located at the orchard, we conclude that relaying on the nearest regional weather station is not a viable option for those farm managers aiming of a precise use of resources such as pesticides or water.

## Supporting information

S1 Fig Images of in-orchard weather stations in different crops (A: Pistachio, B: Almond and C: Apple).
(TIF)

S1 File Relationship between the accumulation growing degree day (GDD) and growing degree hours (GDH) with different base and maximum temperatures (Tm and Tb, respectively) during Q1, Q2 and Q3. (PDF)

S2 File Semivariogram model fit for mean temperature, thermal time, chilll accumulation and phenology forecasts during Q1, Q2, Q3 and the chilling period. (PDF)

S3 File (ZIP)

S1 Data Mean temperature, thermal time, chilll accumulation and phenology forecasts extracted from in-orchard and CIMIS weather stations at each location. (XLSX)
