# Supplementary figures and images for: High-resolution weather network reveals a high spatial variability in air temperature in the Central valley of California with implications for crop and pest management
Source: PLoS One. 2022 May 19;17(5):e0267607. doi: 10.1371/journal.pone.0267607 (PMC9119484; doi:10.1371/journal.pone.0267607)

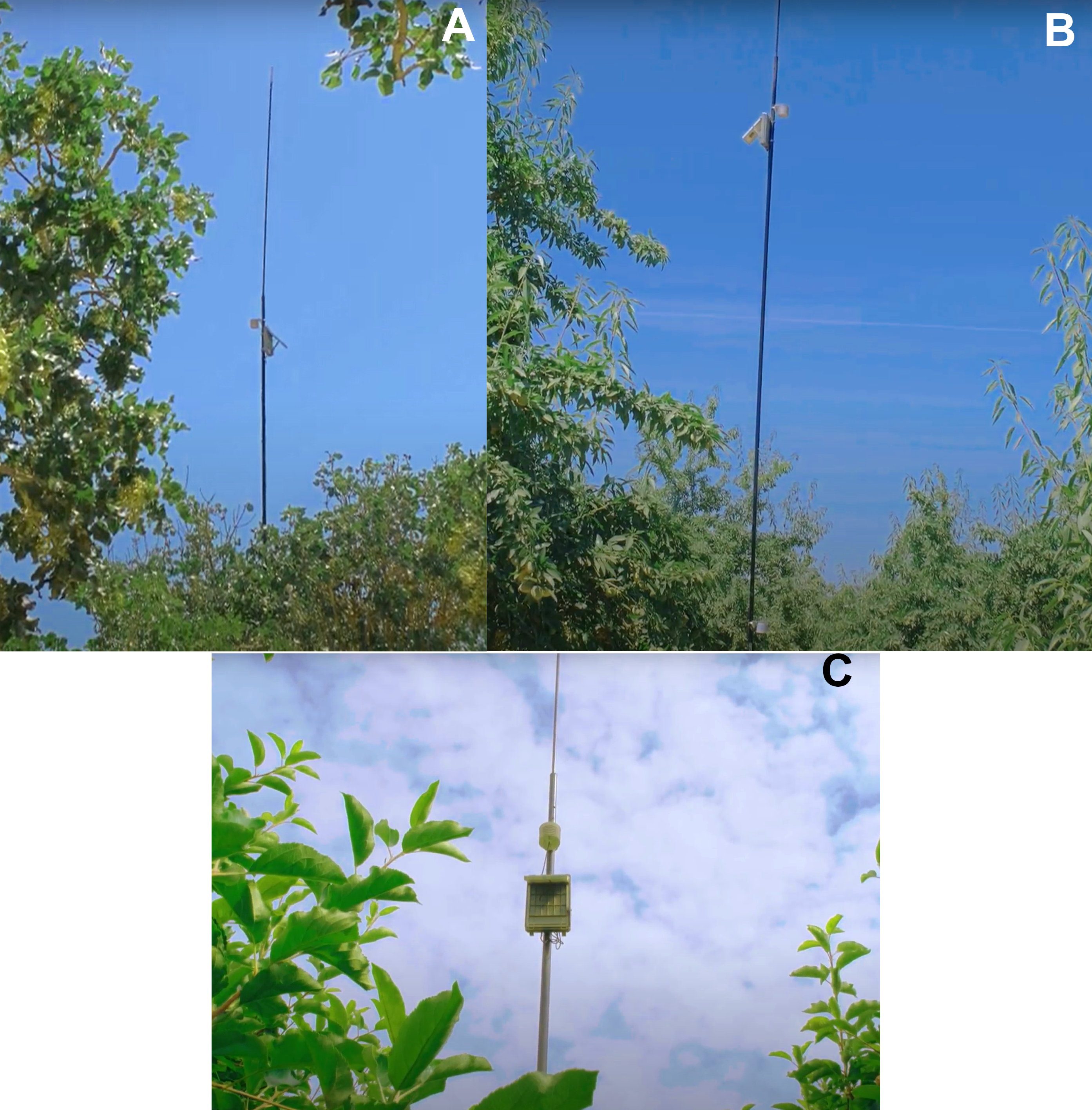

Supplement: S1 Fig — Images of in-orchard weather stations in different crops (A: Pistachio, B: Almond and C: Apple). (TIF) [file pone.0267607.s001.tif]
