# Supplementary material for: High-resolution weather network reveals a high spatial variability in air temperature in the Central valley of California with implications for crop and pest management
Source: PLoS One. 2022 May 19;17(5):e0267607. doi: 10.1371/journal.pone.0267607 (PMC9119484; doi:10.1371/journal.pone.0267607)

Supplementary figure 1. Relationship between the accumulation growing degree day (GDD) and growing degree hours (GDH) with different base and maximum temperatures (Tm and Tb, respectively) during Q1.

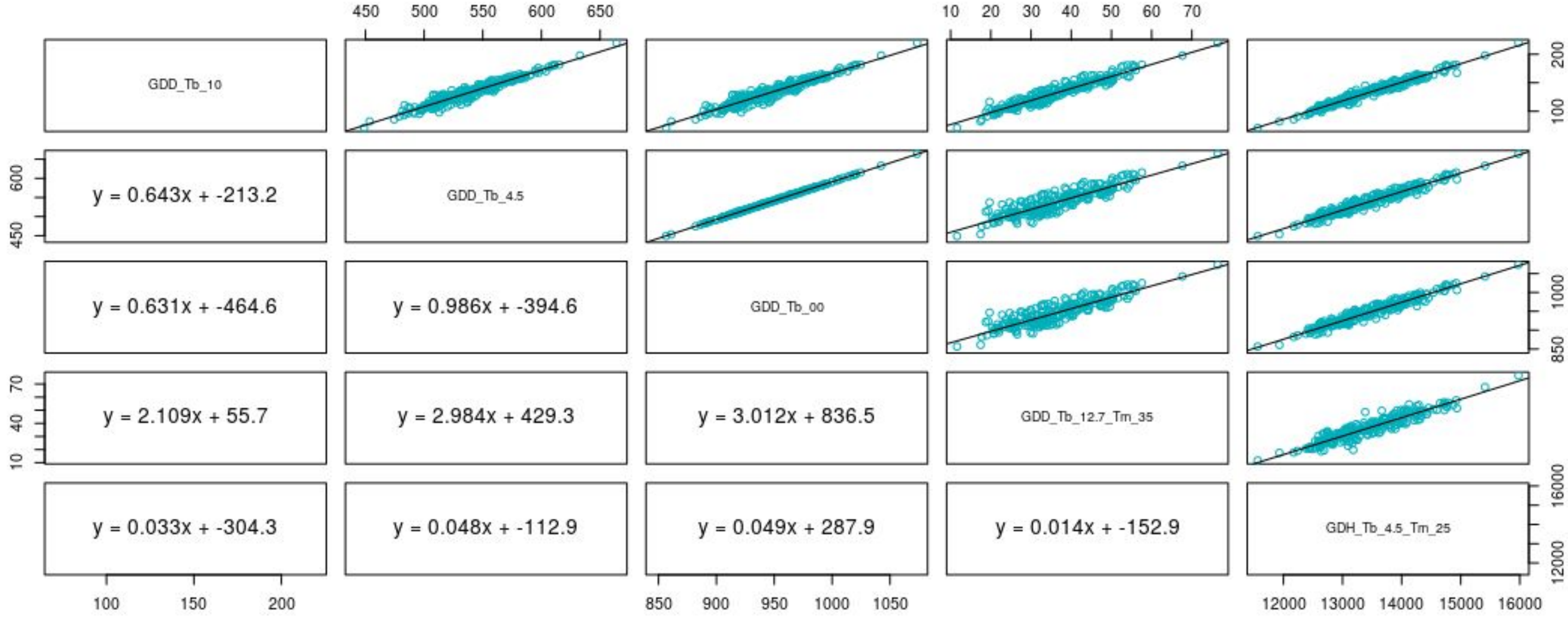

Supplementary figure 2. Relationship between the accumulation growing degree day (GDD) and growing degree hours (GDH) with different base and maximum temperatures (Tm and Tb, respectively) during Q2.

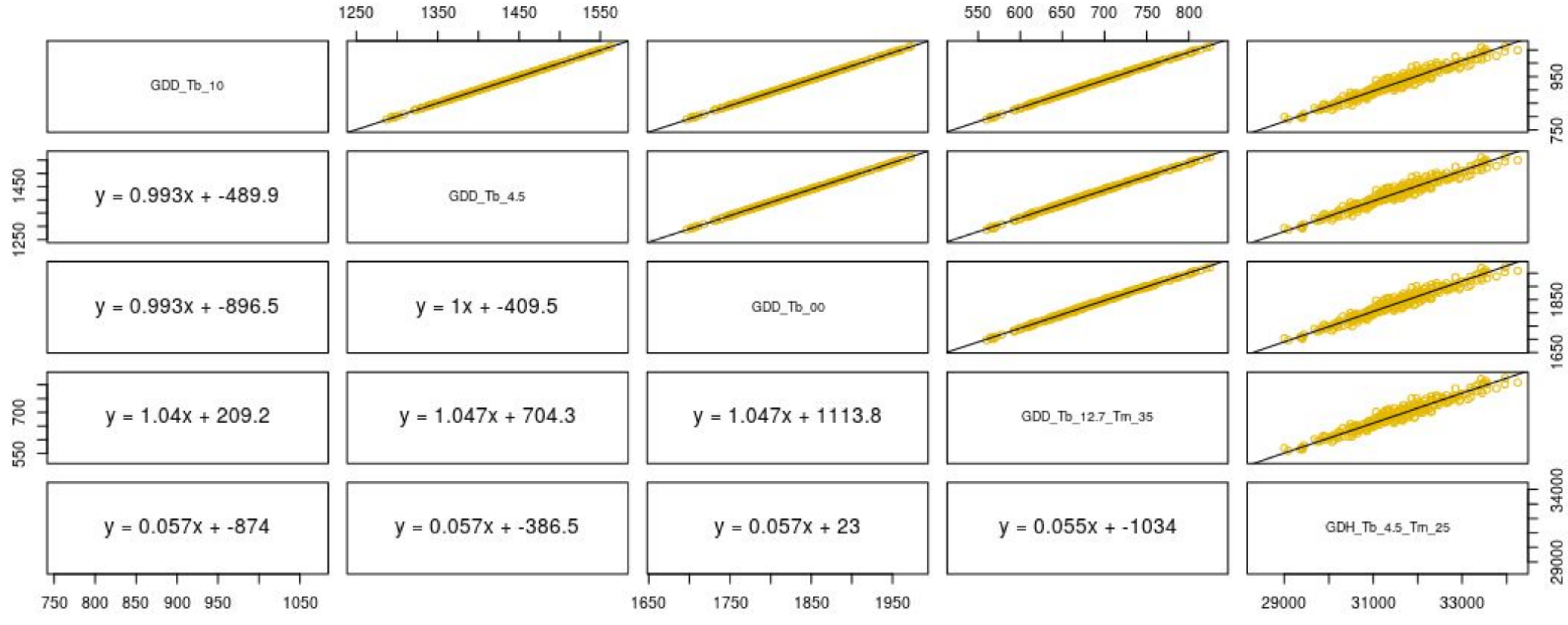

Supplementary figure 3. Relationship between the accumulation growing degree day (GDD) and growing degree hours (GDH) with different base and maximum temperatures (Tm and Tb, respectively) during Q3.

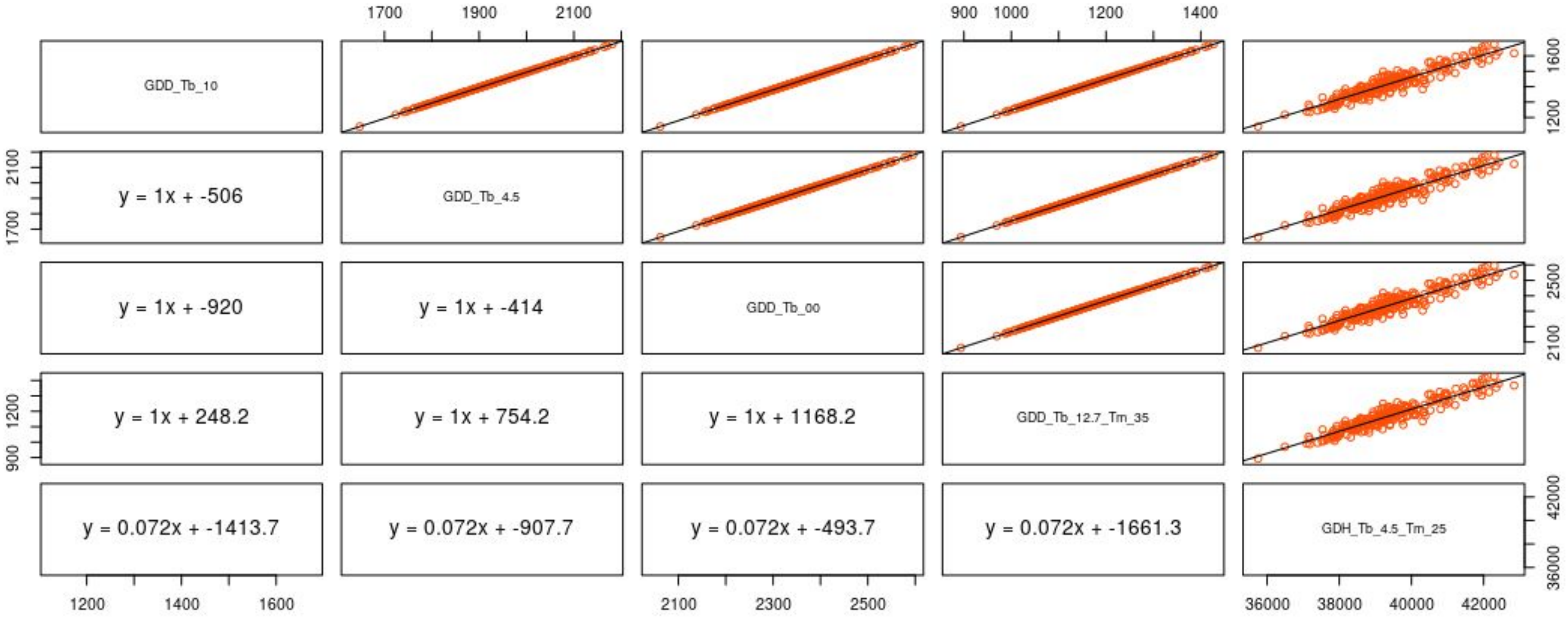

Supplement: S1 File — (PDF) [file pone.0267607.s002.pdf]
