# Supplementary material for: High-resolution weather network reveals a high spatial variability in air temperature in the Central valley of California with implications for crop and pest management
Source: PLoS One. 2022 May 19;17(5):e0267607. doi: 10.1371/journal.pone.0267607 (PMC9119484; doi:10.1371/journal.pone.0267607)

Mean Temperature

Q1: Temperature

All stations

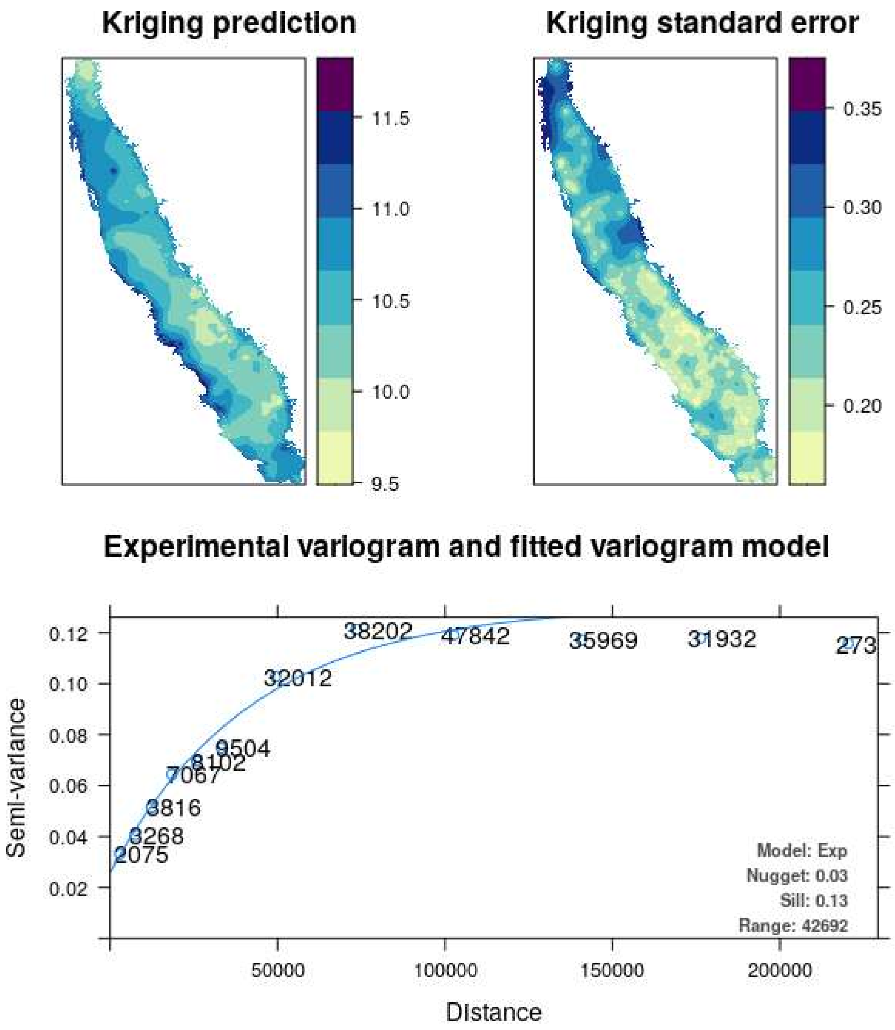

Only CIMIS

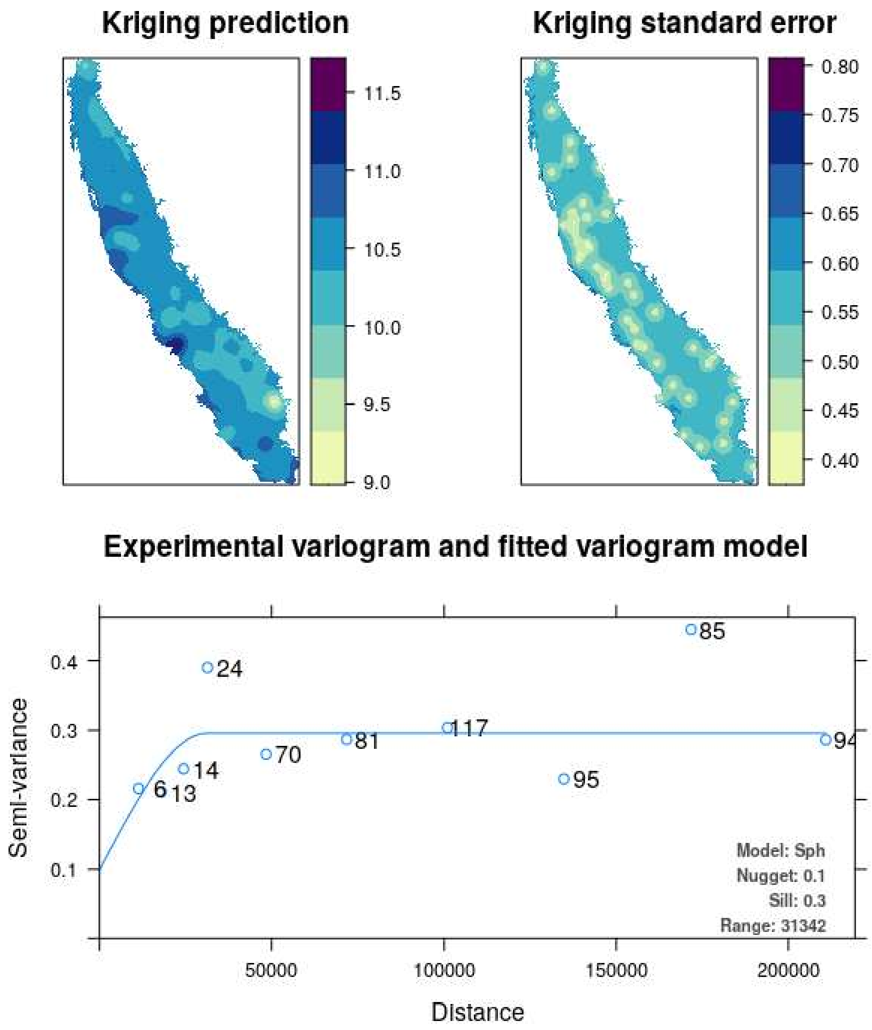

Q2: Temperature

All stations

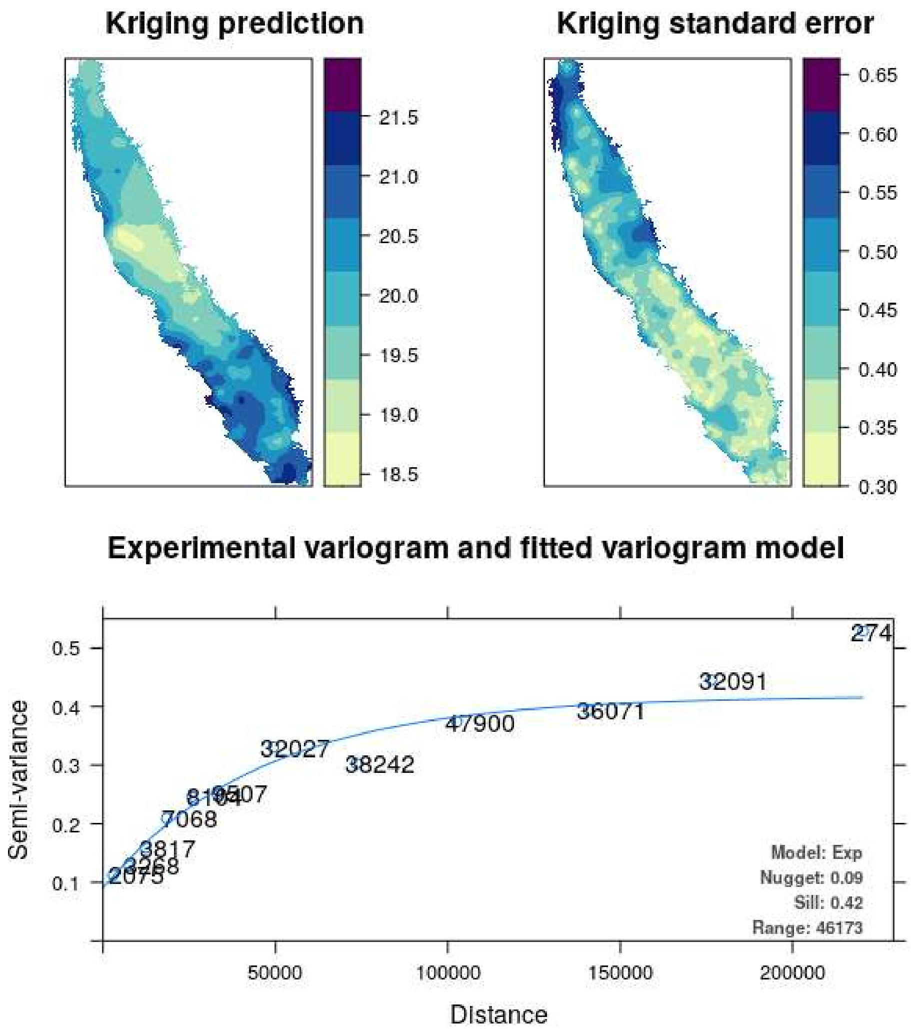

Only CIMIS

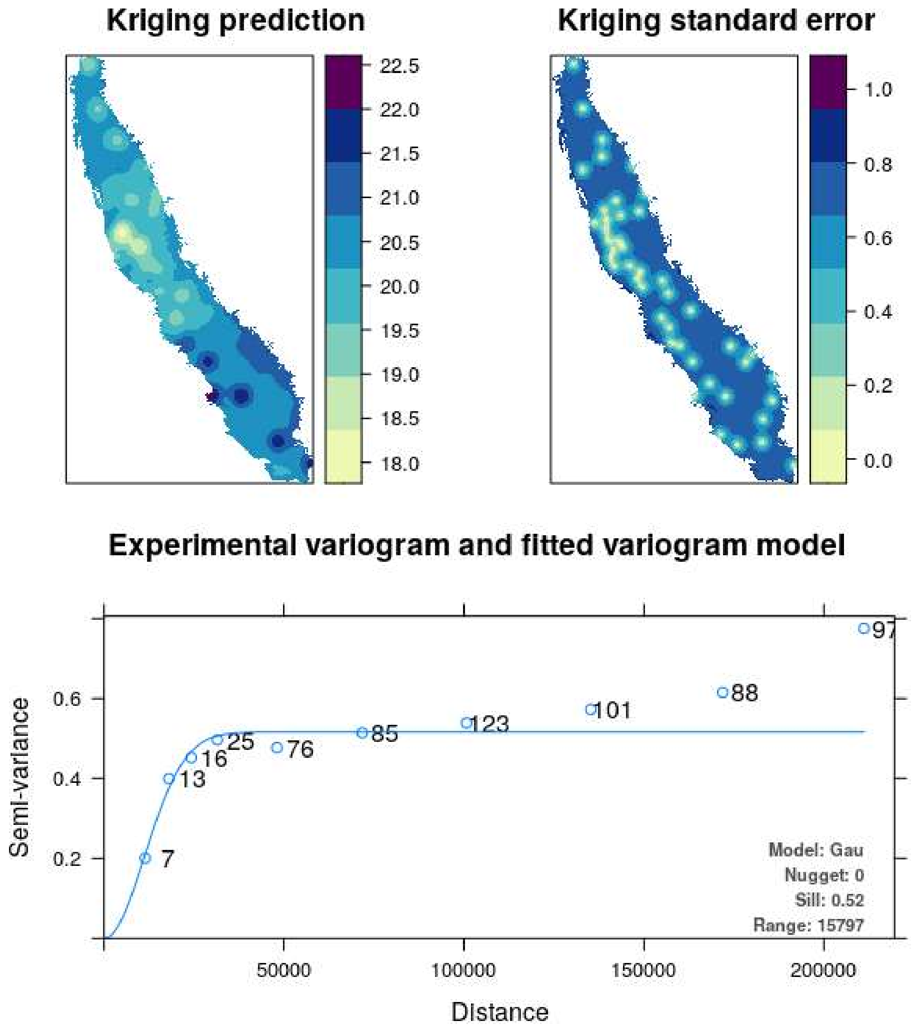

Q3: Temperature

All stations

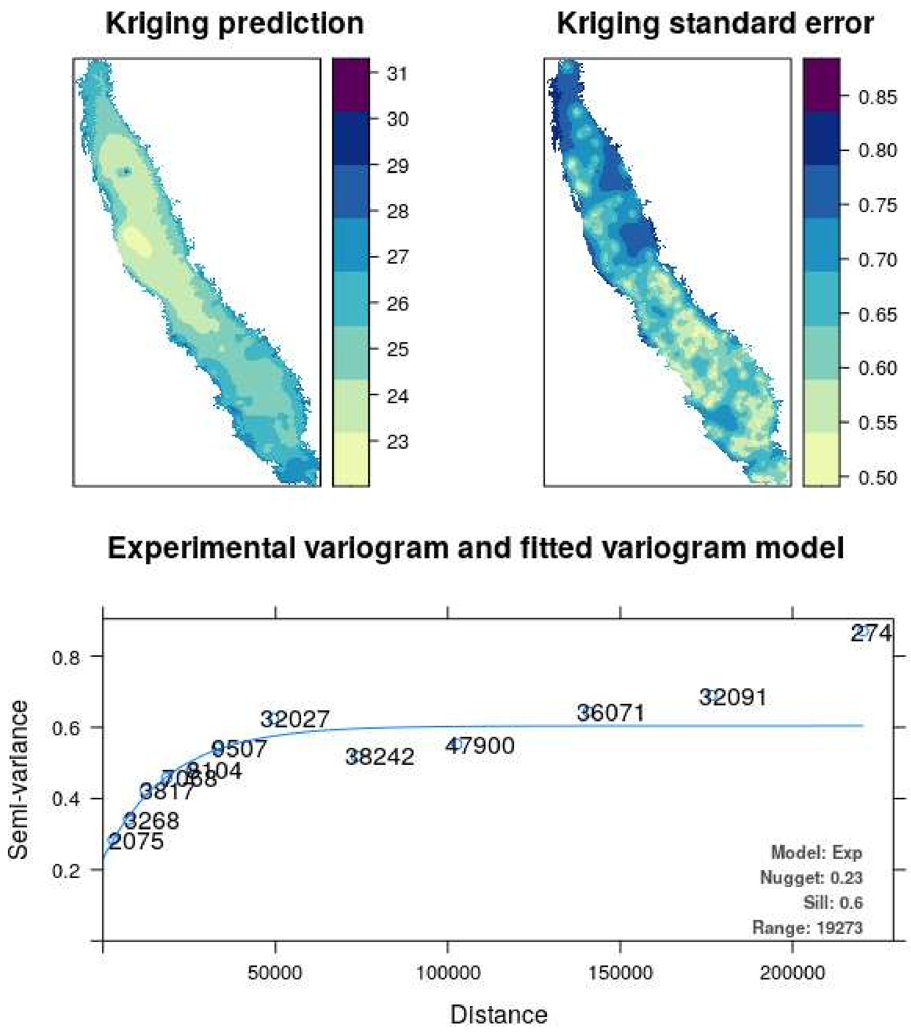

Only CIMIS

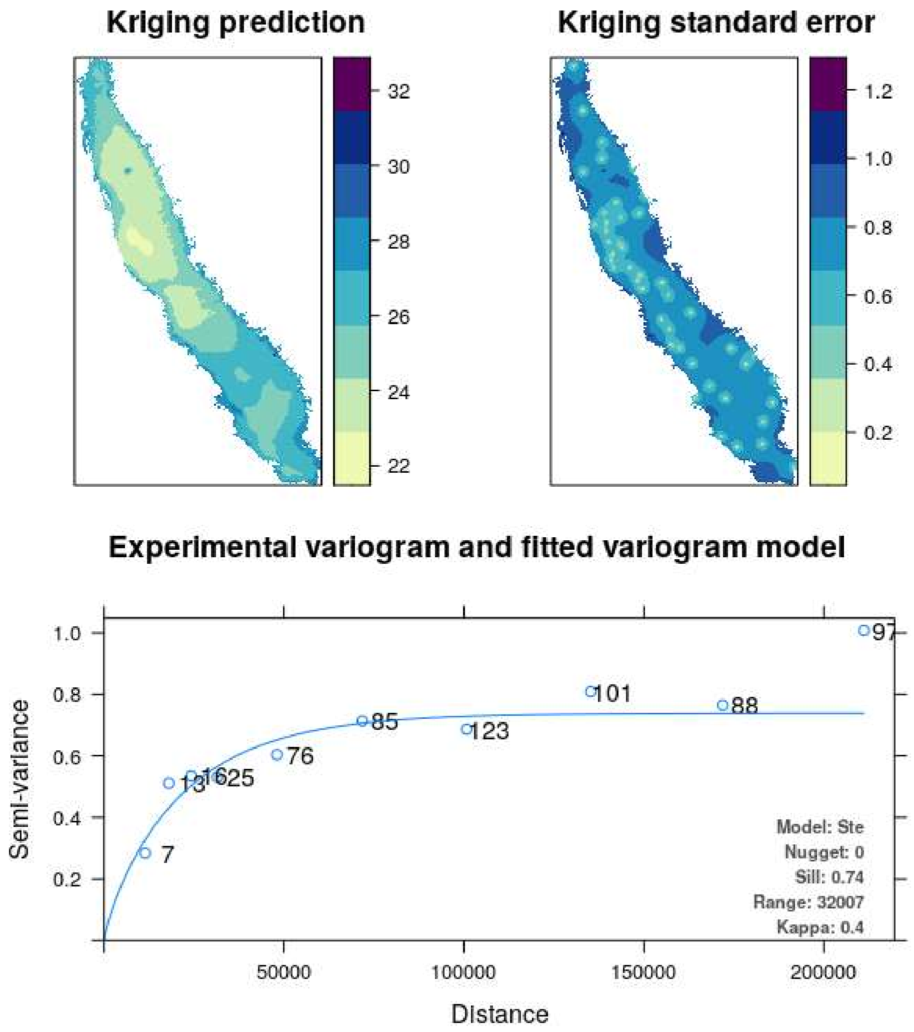

Chill period: Temperature

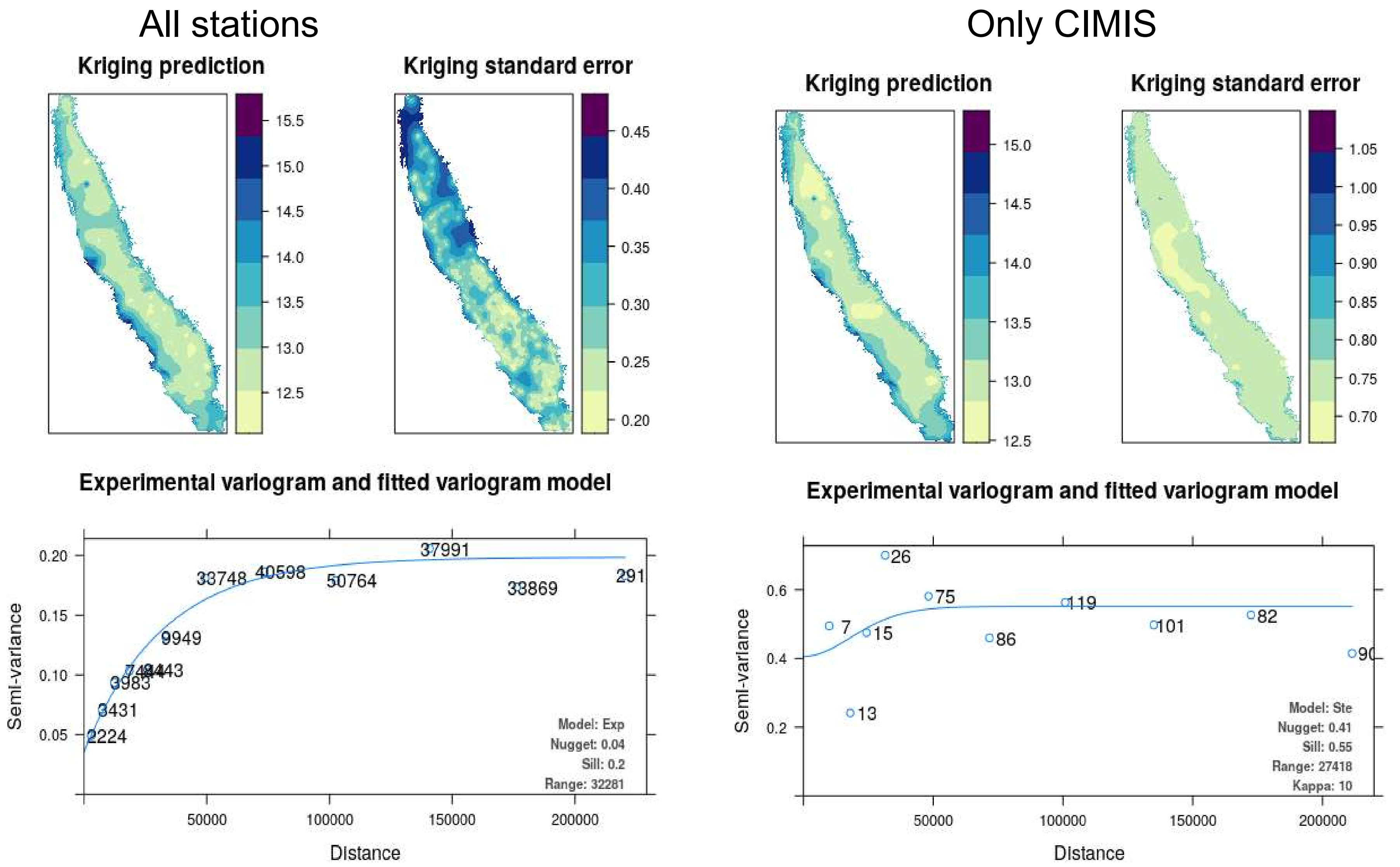

Growing degree days and Chill Portions

Q1: GDD

All stations

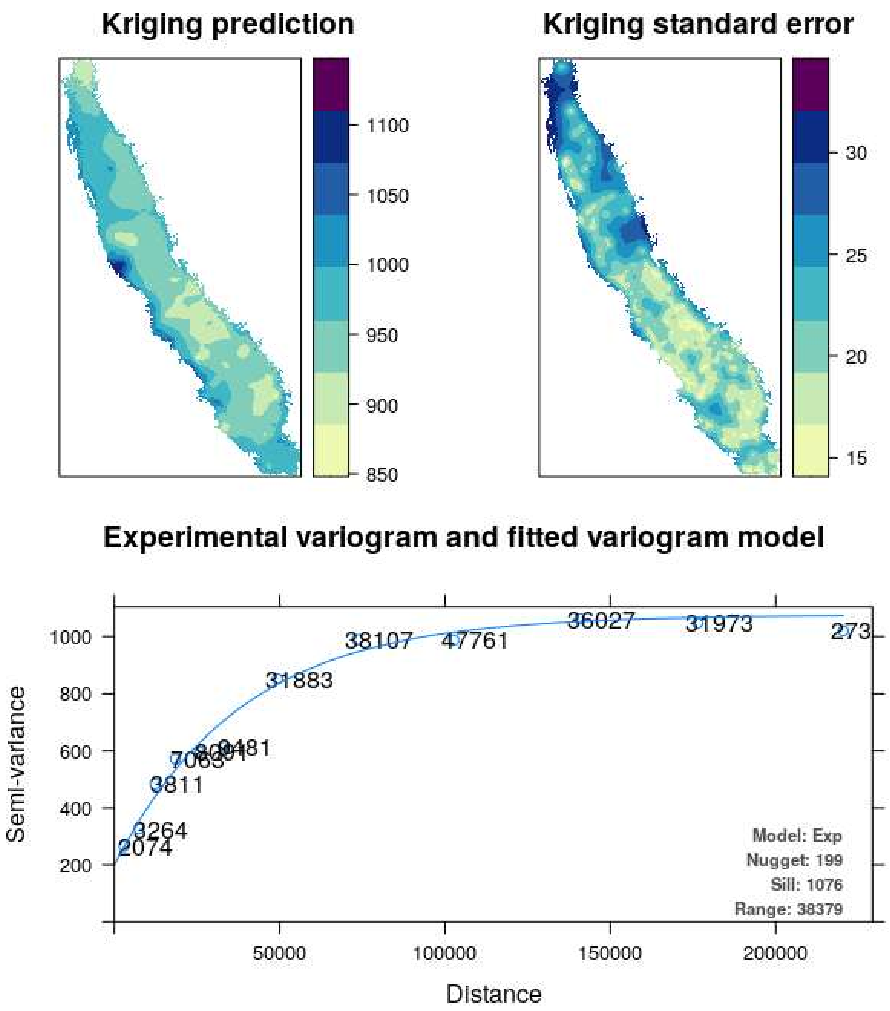

Only CIMIS

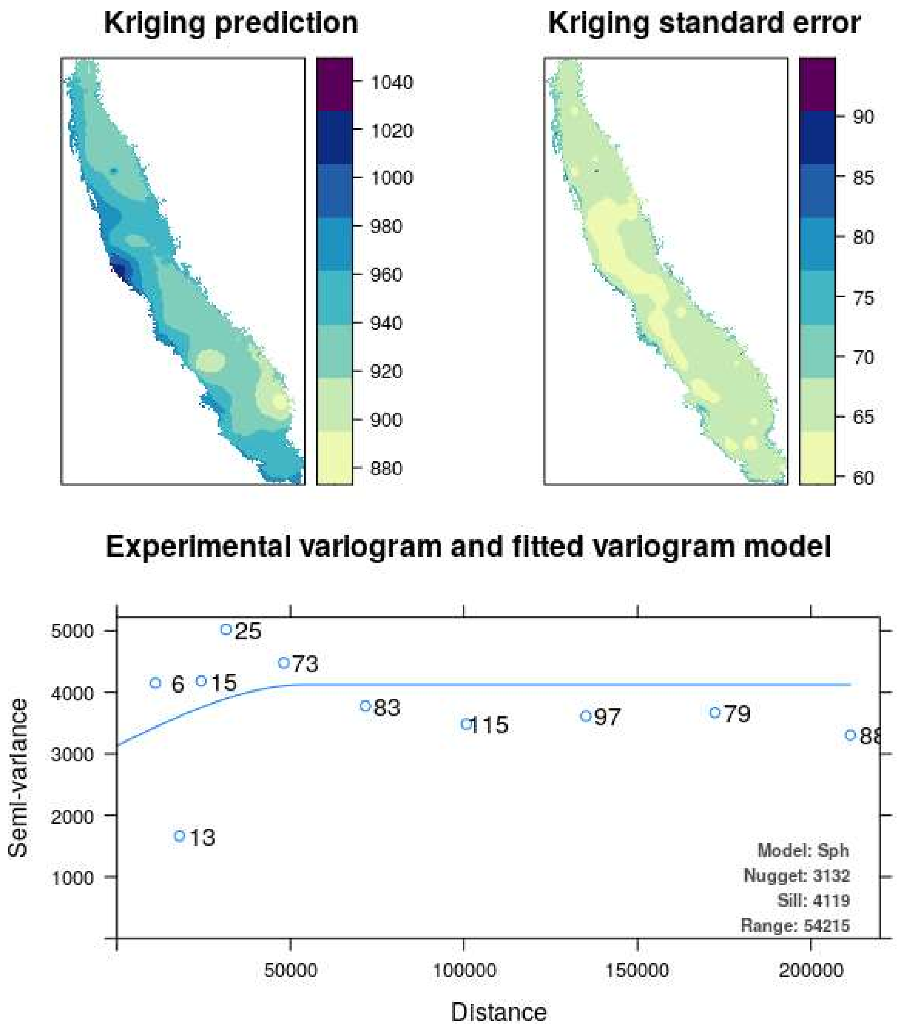

Q2: GDD

All stations

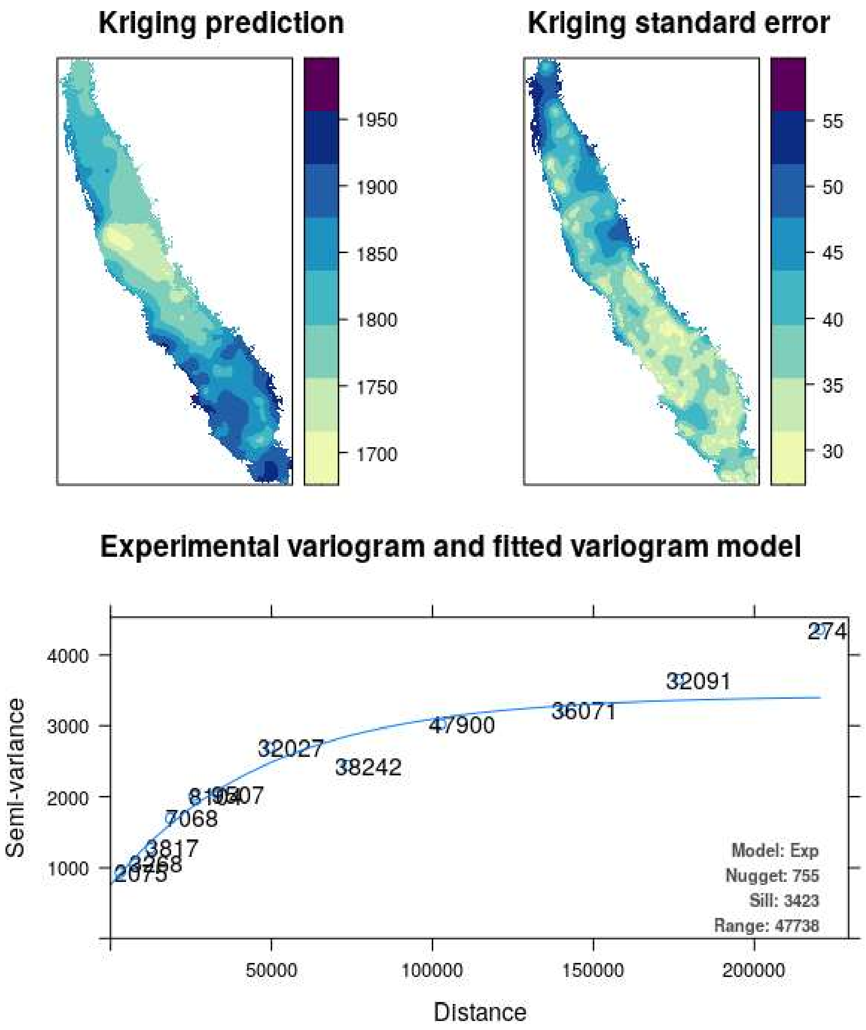

Only CIMIS

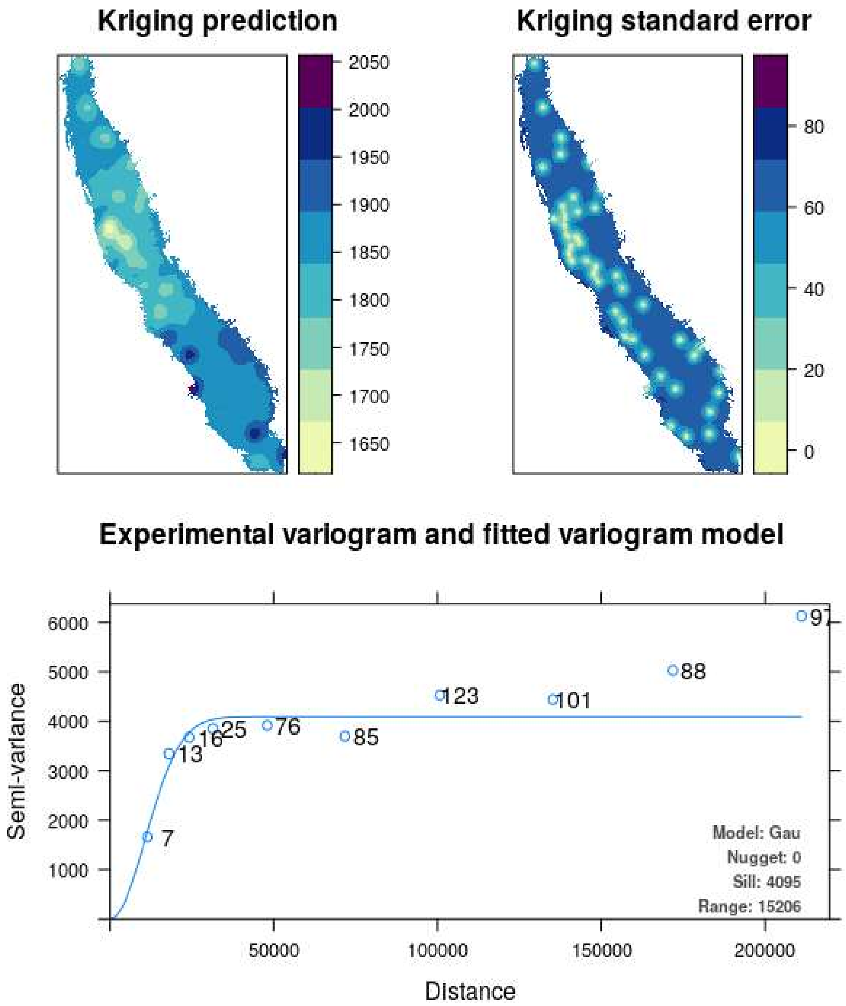

Q3: GDD

All stations

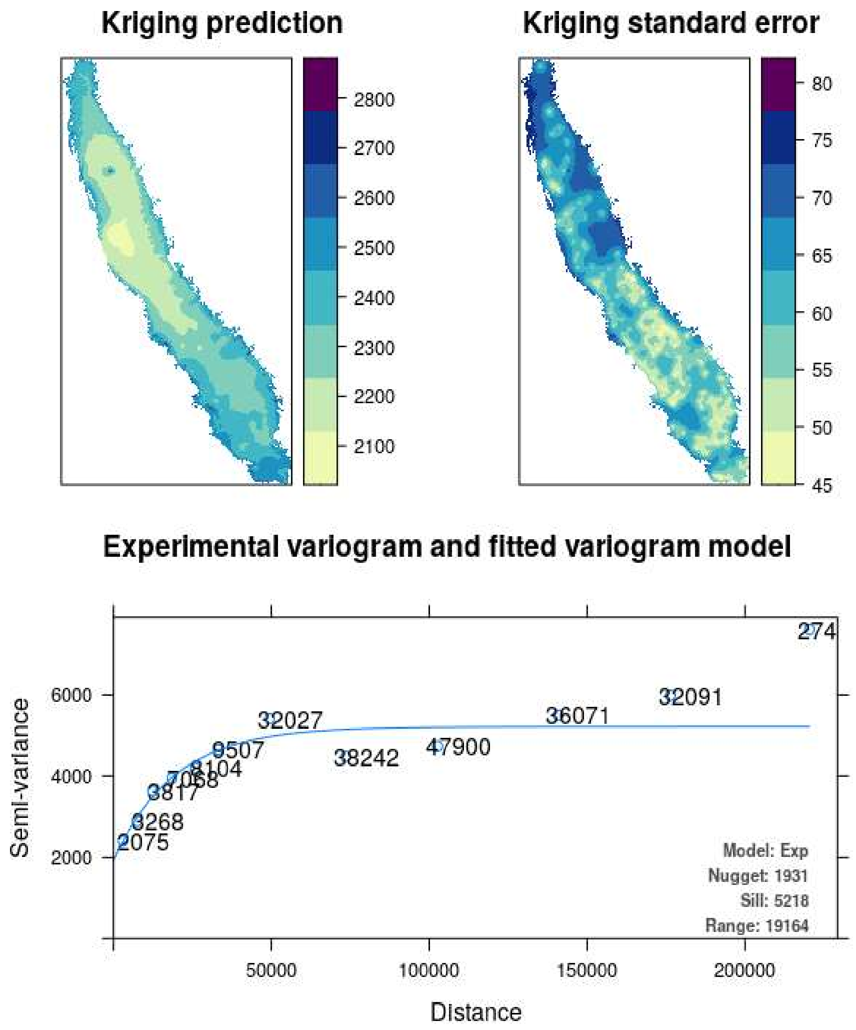

Only CIMIS

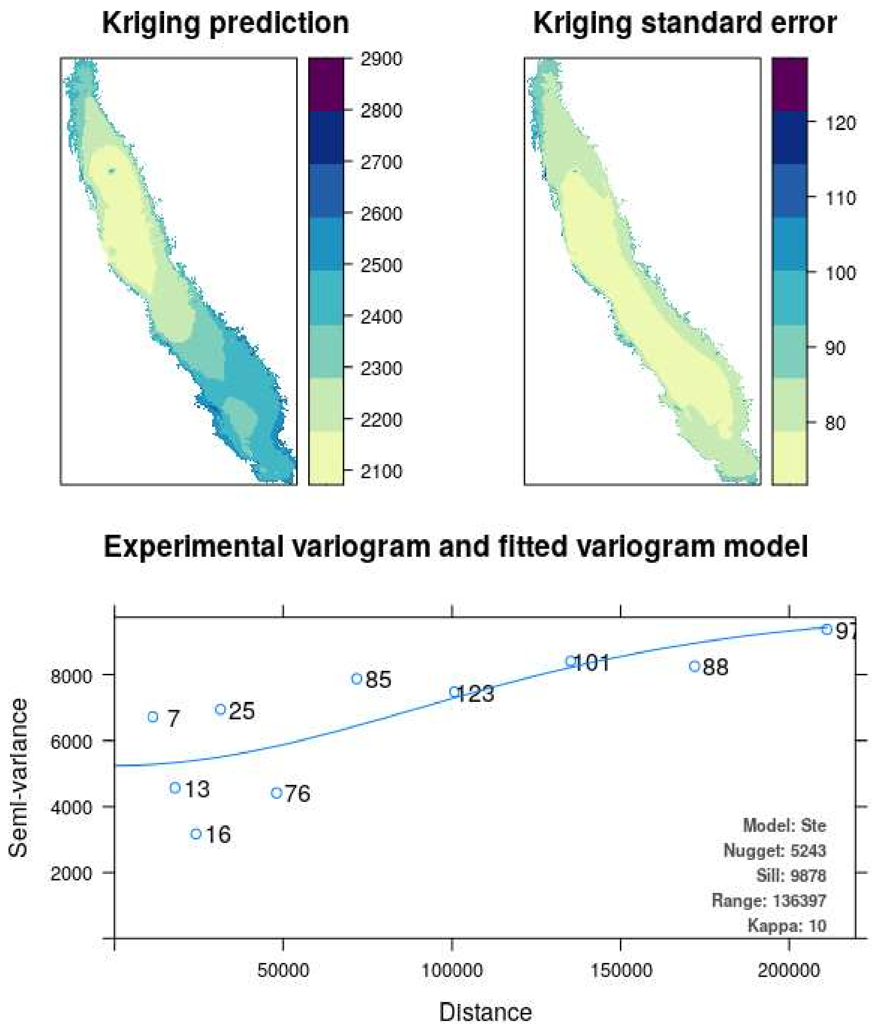

Chill period: CP

All stations

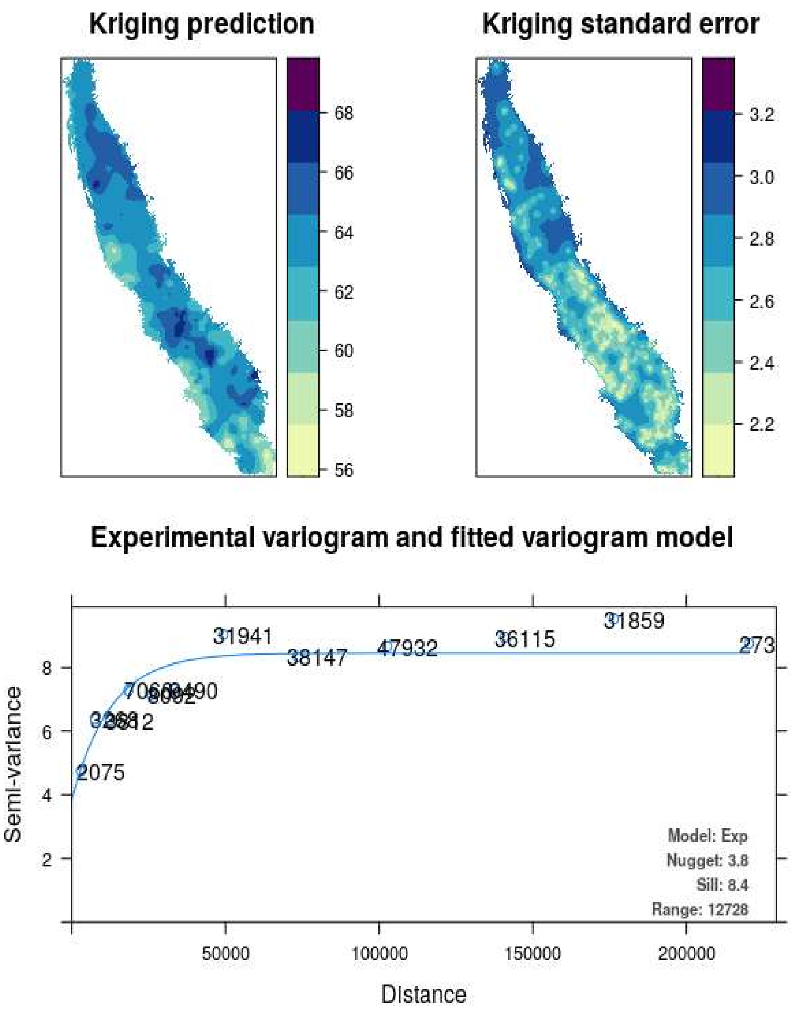

Only CIMIS

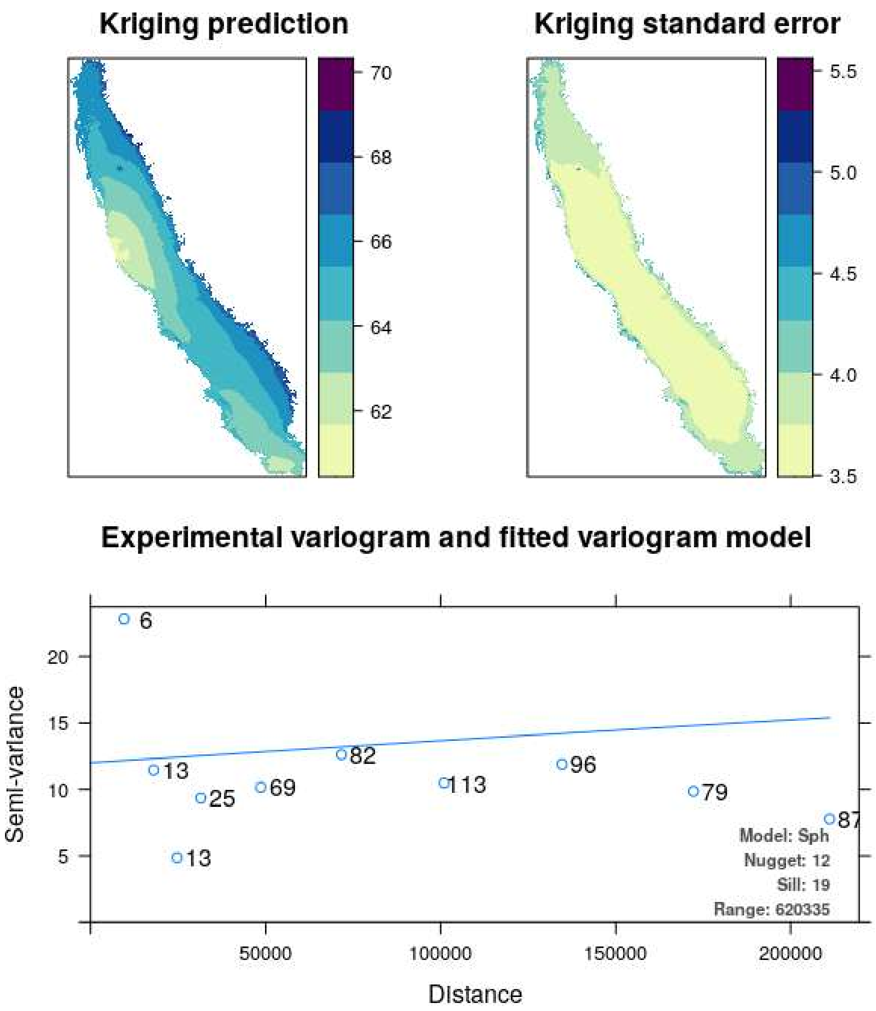

Crop and Pest Phenology

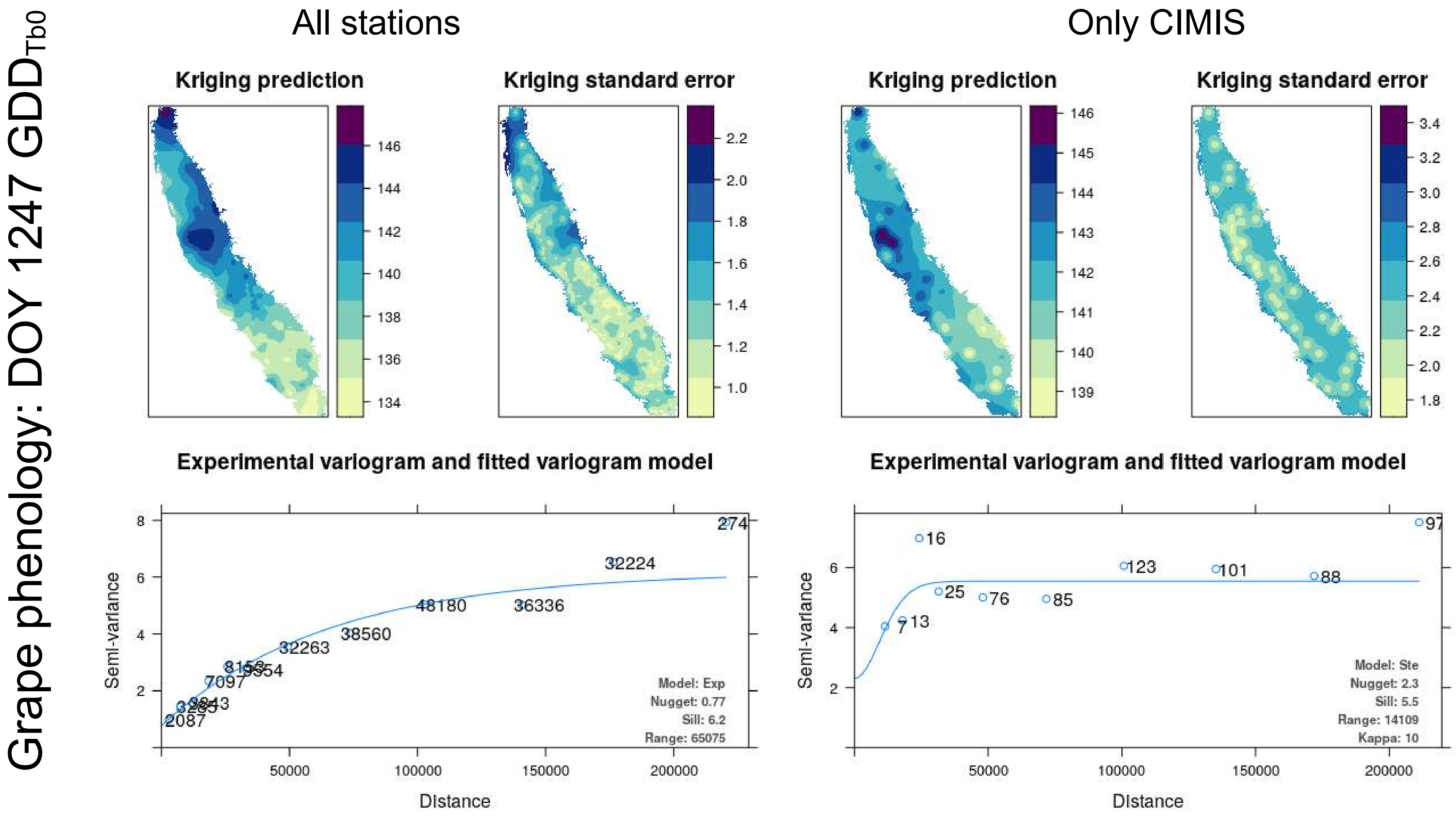
Grape phenology: DOY 1247 GDDTb0
All stations
Only CIMIS
Kriging prediction
Kriging standard error
Kriging prediction
Kriging standard error
Experimental variogram and fitted variogram model
Experimental variogram and fitted variogram model
Semi-variance
Distance
Model: Exp
Nugget: 0.77
Sill: 6.2
Range: 65075
Model: Ste
Nugget: 2.3
Sill: 5.5
Range: 14109
Kappa: 10

Grape phenology: DOY 2547 GDD_{Tb0}

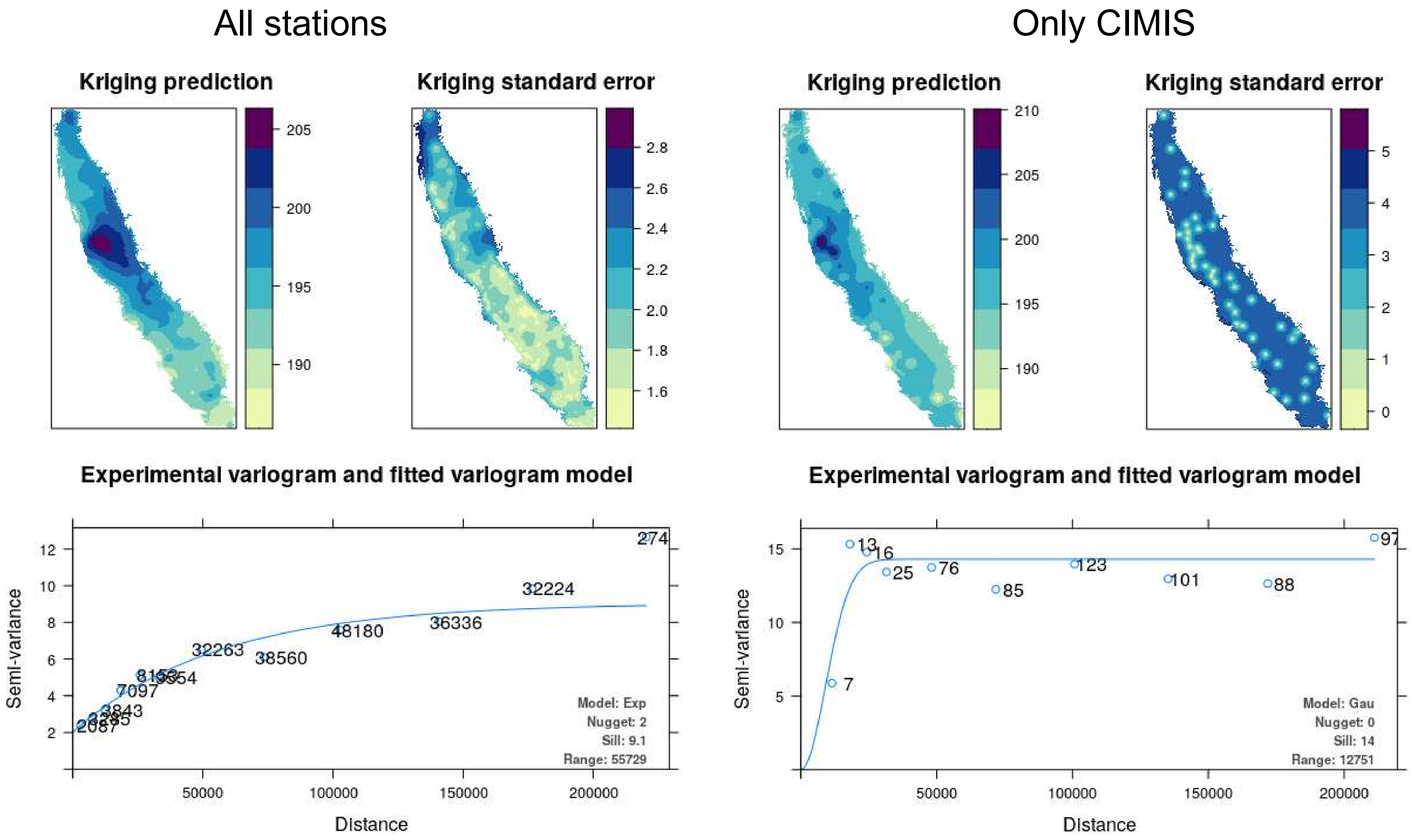

Almond phenology: full bloom

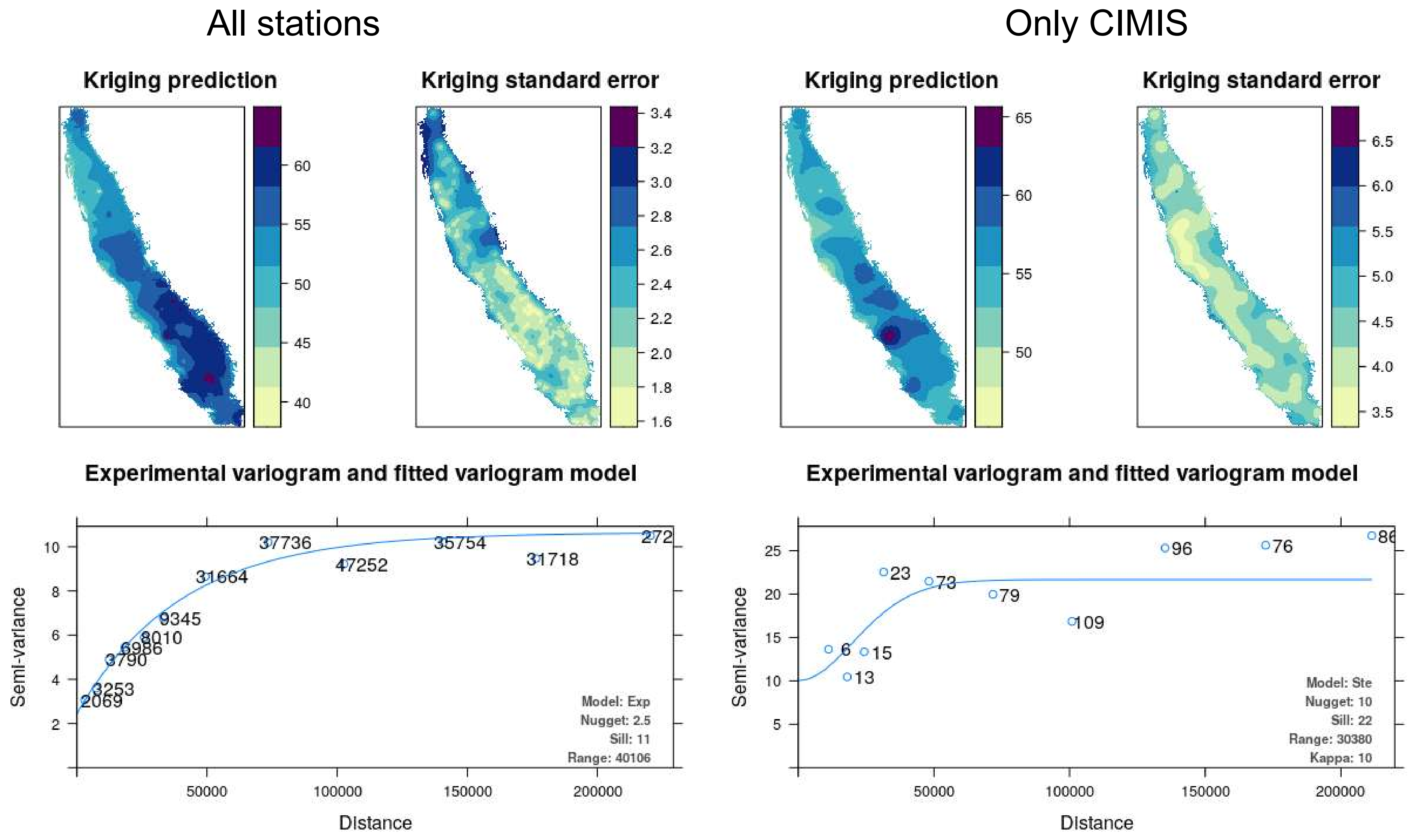

Almond phenology: 1% Hull Split

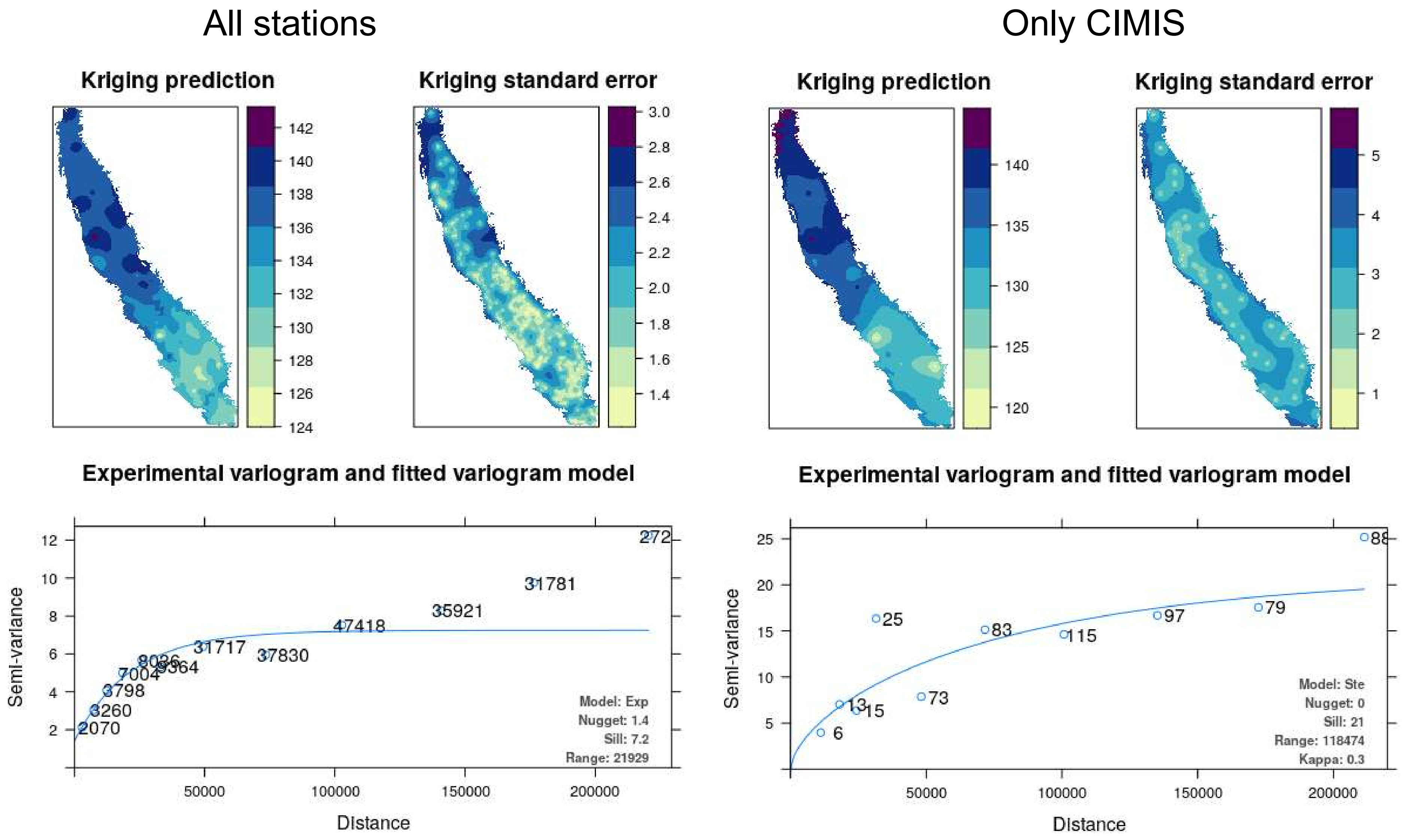

NOW phenology: # generations

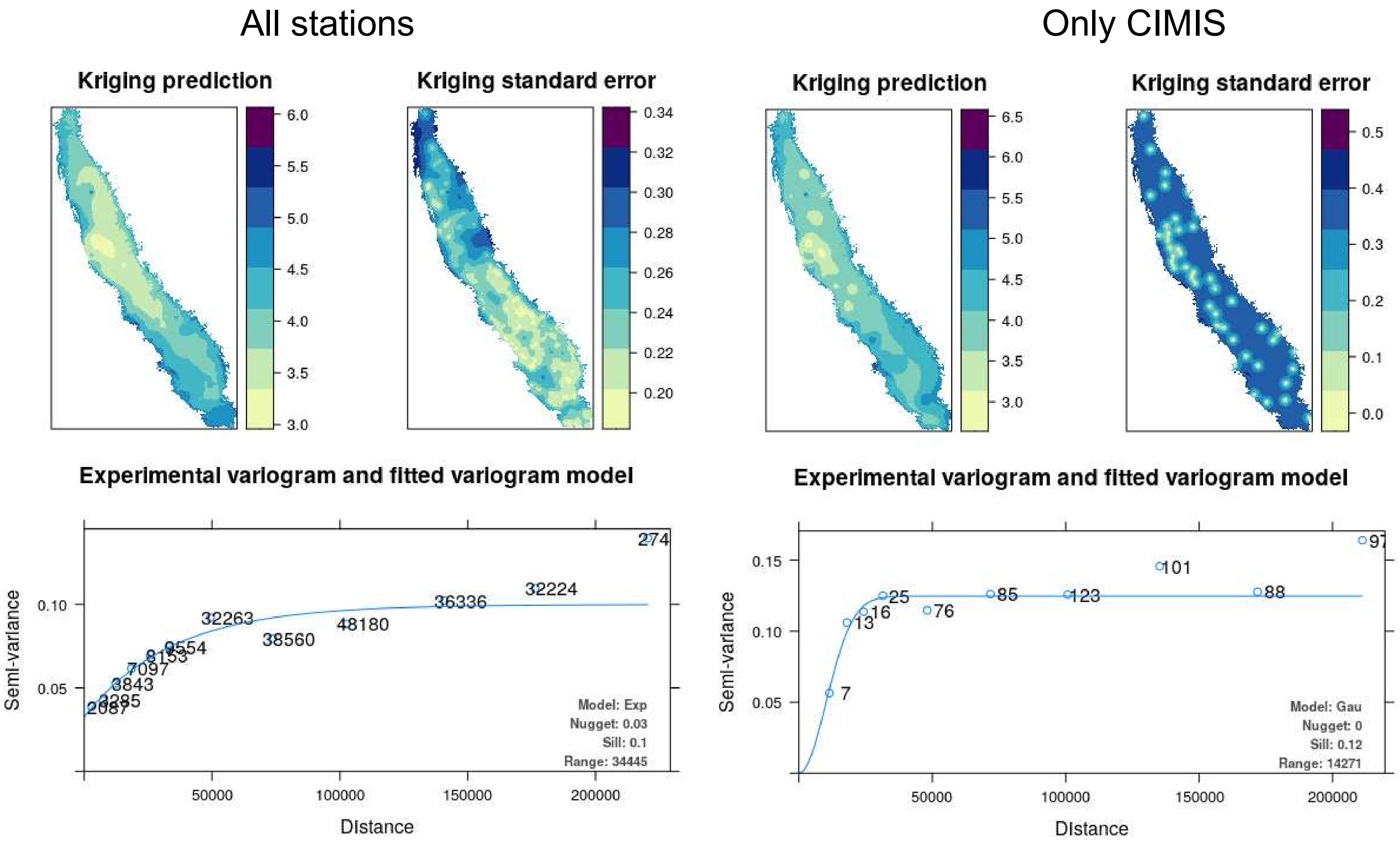

NOW phenology: $GDD_{Tb12.7\_Tm35}$

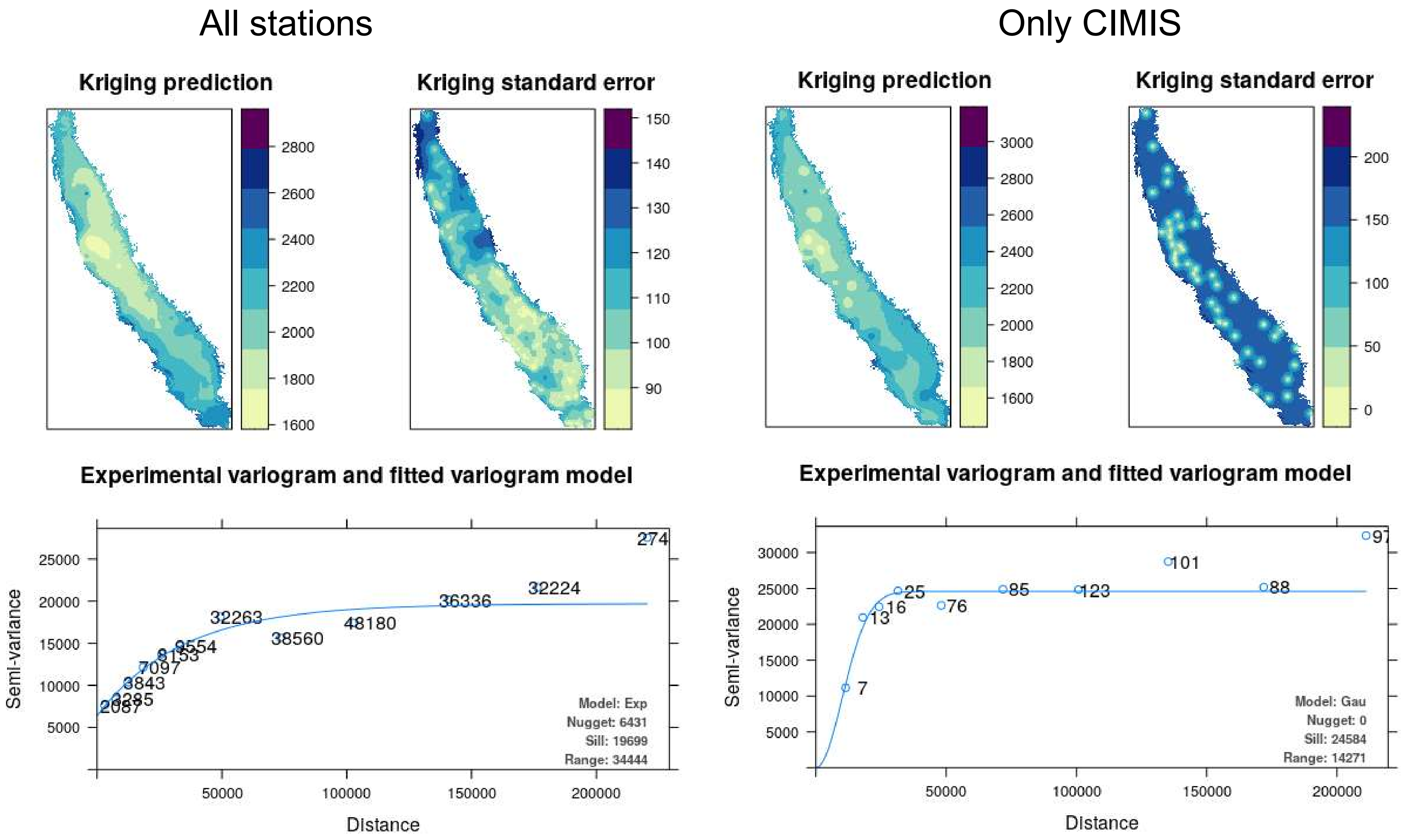

Supplement: S2 File — (PDF) [file pone.0267607.s003.pdf]
